# Differentially Expressed MicroRNAs in Meningiomas Grades I and II Suggest Shared Biomarkers with Malignant Tumors

**DOI:** 10.3390/cancers8030031

**Published:** 2016-03-03

**Authors:** Mohamed Raafat El-Gewely, Morten Andreassen, Mari Walquist, Anita Ursvik, Erik Knutsen, Mona Nystad, Dag H. Coucheron, Kristin Smistad Myrmel, Rune Hennig, Steinar D. Johansen

**Affiliations:** 1 Department of Medical Biology, Faculty of Health Sciences, UiT-The Arctic University of Norway, NO-9037 Tromsø, Norway; morten.andreassen@gmail.com (M.A.); mari.walquist@uit.no (M.W.); anita.ursvik@uit.no (A.U.); erik.knutsen@uit.no (E.K.); dag.coucheron@uit.no (D.H.C.); steinar.johansen@uit.no (S.D.J.); 2 Department of Clinical Medicine, Women’s Health and Perinatology Research Group, Faculty of Health Sciences, UiT-The Arctic University of Norway, NO-9037 Tromsø, Norway; mona.nystad@unn.no; 3 Department of Obstetrics and Gynecology, University Hospital of North Norway, NO-9038 Tromsø, Norway; 4 Department of Medical Genetics, Division of Child and Adolescent Health, University Hospital of North Norway, NO-9038 Tromsø, Norway; 5 Department of Pathology, University Hospital of North Norway, NO-9038 Tromsø, Norway; kristin.smistad.myrmel@unn.no; 6 Department of Neurosurgery, University Hospital of North Norway, NO-9038 Tromsø, Norway; rune.otto.hennig@unn.no; 7 Department of Clinical Medicine, Division of Neurosurgery, Faculty of Health Sciences, UiT-The Arctic University of Norway, NO-9037 Tromsø, Norway; 8 Marine Genomics Group, Faculty of Biosciences and Aquaculture, Nord University NO-8049 Bodø, Norway

**Keywords:** meningioma, SOLiD deep sequencing, miRNA, RT-qPCR, cap cells and Immunohistochemistry (IHC)

## Abstract

Meningiomas represent the most common primary tumors of the central nervous system, but few microRNA (miRNA) profiling studies have been reported so far. Deep sequencing of small RNA libraries generated from two human meningioma biopsies WHO grades I (benign) and II (atypical) were compared to excess dura controls. Nineteen differentially expressed miRNAs were validated by RT-qPCR using tumor RNA from 15 patients and 5 meninges controls. Tumor suppressor miR-218 and miR-34a were upregulated relative to normal controls, however, miR-143, miR-193b, miR-451 and oncogenic miR-21 were all downregulated. From 10 selected putative mRNA targets tested by RT-qPCR only four were differentially expressed relative to normal controls. *PTEN* and E-cadherin (*CDH1*) were upregulated, but *RUNX1T1* was downregulated. Proliferation biomarker *p63* was upregulated with nuclear localization, but not detected in most normal arachnoid tissues. Immunoreactivity of E-cadherin was detected in the outermost layer of normal arachnoids, but was expressed throughout the tumors. Nuclear Cyclin D1 expression was positive in all studied meningiomas, while its expression in arachnoid was limited to a few trabecular cells. Meningiomas of grades I and II appear to share biomarkers with malignant tumors, but with some additional tumor suppressor biomarkers expression. Validation in more patients is of importance.

## 1. Introduction

Intracranial meningiomas are slow-growing tumors arising from the outer layer of the arachnoid (cap cells) that are non-neuroepithelial. These cap cells are a morphologically distinct and biochemically active subgroup of arachnoidal cells [1]. Meningiomas are the most common (35.6%) of all primary intracranial tumors by histology [2], and they are classified into grades I–III according to the WHO grading system [3]. The proliferation rate in meningioma is known to increase from grades I–III (Table S1a). The majority of clinically encountered meningiomas is benign, and corresponds to grade I. These types of meningiomas have a slow growth rate with low risk of recurrence or a malignant behavior. Despite their common origin, meningiomas present a wide variety of histological and morphological appearances (Table S1b). Most meningiomas harbor at least one of these histological characteristics, but they seldom occur in pure form.

Molecular studies on slowly growing non-malignant meningiomas could add important knowledge about factors underlying tumor formation, as well as factors preventing benign tumors from progression to malignancy. Our current knowledge about tumorigenesis is largely based on the study of malignant tumors while the biology of benign neoplasms is rarely investigated [4]. Currently, only a few reports have addressed the molecular biology of meningioma at the transcriptomic level, mainly using microarray platforms [5,6,7,8]. Germline mutation in the *NF2* gene is the most commonly identified genetic risk factor for multiple meningioma disease [9]. However, in non-*NF2* meningioma, somatic mutations in *TRAF7, KLF4, AKT1* and *SMO* genes were reported [9,10]. Recently it became clear that epigenetic mechanisms such as DNA methylation, histone modifications and expression of microRNAs (miRNAs) play an important role in cancer and contribute to malignant transitions [11,12]. The key epigenetic factors and their role in meningioma initiation, progression and recurrence are recently reviewed [13,14]. miRNAs are short non-coding RNAs of approximately 22 nucleotides, and the current estimate is that 4552 different miRNAs are coded in the human genome[15]. Most miRNAs function by base pairing to the 3′-untranslated region (3′UTR) of targeted mRNAs resulting in protein translation arrest or mRNA degradation via the RNA-induced silencing complex [16]. Dysregulation of miRNA expression or their biogenesis could lead to cancer, and miRNA biogenesis pathways in cancer have recently been reviewed [17,18]. The repression of protein translation by miRNA depends on several factors such as the levels of target mRNA and miRNA expression, the complexity of expressed miRNA that can target the same mRNA, other expressed RNAs, or the physiological condition of the cell [19]. miRNAs and their dysregulation hold great potential as clinical biomarkers of physiological and pathological states in cancer, in development, and in immunological inflammatory reactions [20,21,22,23]. Investigating and comparing expression profiles of miRNAs in non-malignant tumors with those of malignant tumors would help clarify their role in tumorigenesis and growth, as well as in preventing non-malignant tumors from progressing to malignancy. Here only a limited number of miRNAs were investigated, but interesting differentially expressed candidates were detected.

In the presented work we focused on non-malignant meningiomas grade I (benign) and grade II (atypical) in order to investigate differentially expressed miRNAs by SOLiD deep sequencing of tumors and excess dura from the same two patients (N), in addition to two patients without meningioma (NN). Differentially expressed miRNAs was further assessed by RT-qPCR using tumors from fifteen patients and five control tissues (dura), one of which was from a patient without meningioma. A selection of ten putative mRNA targets was then evaluated by RT-qPCR in tumors of 15 patients relative to five dura controls using *GAPDH* reference gene. RT-qPCR was subsequently repeated for five promising targets, in addition to E-cadherin (*CDH1*) for all the 15 patient tumor samples and four dura controls, using three reference genes (*GAPDH*, *ACTB* (β-Actin) and *HPRT*). Immunohistochemistry (IHC) was used to further examine the expression of key differentially expressed genes from tumors (grades I and II) and normal dura and arachnoid tissues.

## 2. Results

### 2.1. Pathological and Histological Classifications

**Figure 1:**
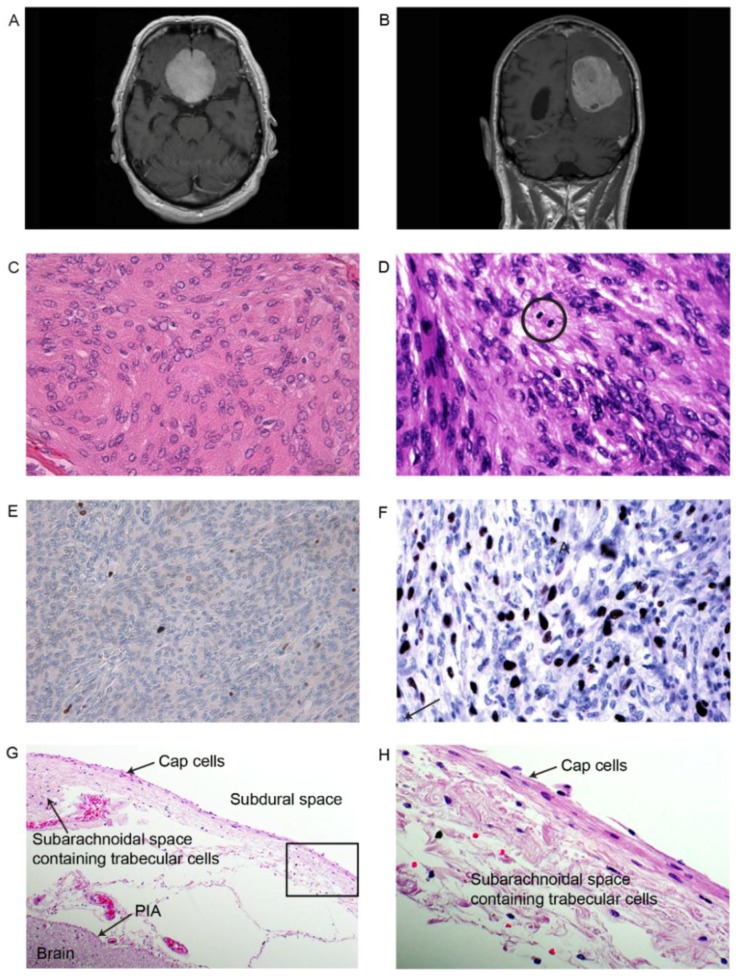
Tumors of the two patients used for sequence analysis. Tumor from patient 1 (IA) presented to the left (**A**, **C** and **E**), and tumor from patient 2 (II) to the right (**B**, **D** and **F**); (**A**) and (**B**) show the Magnetic resonance imaging (MRI) for patients 1 and 2 (Table 1); (**C**) Meningothelial meningioma grade I with lobules of uniform meningioma cells with typical intranuclear inclusions. Hematoxylin and eosin stain (HE), 400×; (**D**) Atypical meningioma grade II with atypical features and scattered mitoses. HE, 600×; (**E**) Very few immunoreactive tumor cells for the proliferation marker Ki-67 in this grade I tumor, 200×; (**F**) Elevated activity for the proliferation marker Ki-67 in this grade II meningioma (200×); (**G**) The normal arachnoid contains an external layer of horizontally oriented cap cells (200×). Traversing the subarachnoid, a loose web-like tissue containing trabecular cells, fibrous tissue and vessels that fuse with the inner Pia mater covering the brain surface; (**H**) The enlarged marked section in G at 400× showing the horizontally oriented external cap cells in the arachnoid membrane.

Meningiomas removed from patient 1 (grade I, subgroup A) and patient 2 (grade II) (Figure 1) demonstrated different histological features. The IA tumor had histology of a classical meningothelial meningioma with low cellularity, lobular growth pattern containing uniform cells, and showed no signs of atypia. The nuclei were oval, occasionally with clear intranuclear inclusions (Figure 1C). Necrosis or mitosis was not found, nor invasion of brain tissue. The proliferation marker Ki-67 was low, close to 2% (Figure 1E). The tumor was diagnosed as a meningioma WHO grade I, and morphologically further classified into the meningothelial subgroup (Table S1a,b).

The pathological specimen from the grade II tumor from patient 2 harbored several atypical criteria that included a higher cellularity. Atypia with pleomorphic nuclei and prominent nucleoli were seen, as well as foci of necrosis. Scattered mitosis appeared, though not four or more per 10× 40× magnification (Figure 1D). The expression of proliferation marker Ki-67 was elevated, focally close to 10%, supporting the diagnosis of an atypical meningioma (Figure 1F). The diagnosis concluded an atypical meningioma WHO grade II. Histological examination of normal dura (NN) revealed fibrous tissue consistent with dura, containing a small focus of arachnoid cells, morphologically consistent with cap cells. The specimens from the arachnoid contained normal arachnoid covering with an outer layer of horizontally oriented cap cells, and an inner looser part containing trabecular cells traversing the sub-arachnoid space, as shown in Figure 1G.

### 2.2. MiRNA Expression Profile from SOLiD Deep Sequencing

Libraries of small RNAs from tumors (IA and II), normal dura (N) outside tumors of same patients, and normal dura (NN) of patients without meningioma (Table 1) were deep sequenced. Comparing the two types of controls by using the complete set of detected miRNAs suggest no significant differences in expression at the global level (Table S2c). From the analysis of the small RNA sequencing (Table S2a,b), differentially expressed miRNAs with at least five folds change (Table 2), were selected for re-examination by RT-qPCR.

### 2.3. RT-qPCR Re-Evaluation of Differentially Expressed miRNAs in Meningioma Versus Normal

The top19 differentially expressed miRNAs from SOLiD deep sequencing (Table 2) were examined by RT-qPCR in all 15 meningioma tumor samples and five dura controls N and NN (Table 1 and Table S3a). Human miR-191, miR-16 and let-7a were used as reference miRNA genes [42] for normalization. A fold change of −81.56 between the grade II meningioma tumor sample and the normal sample (Table 2) for miR-122 was identified by deep-sequencing. However, this feature was not found in any of the other tumor samples by a preliminary test RT-qPCR and thus excluded from further analysis. The miRNA validation results for the 18 miRNAs are listed in Table 3 and Table S3a. miRNA expression with fold change values above three relative to normal dura (N and NN), are given as ΔCq values with Standard deviation in Figure S1. The RT-qPCR data for these miRNAs did not show any significant difference between grades I and II (Table S3b).

### 2.4. RT-qPCR Evaluation of Expression of Selected mRNA Targets in Meningiomas

**Figure 2:**
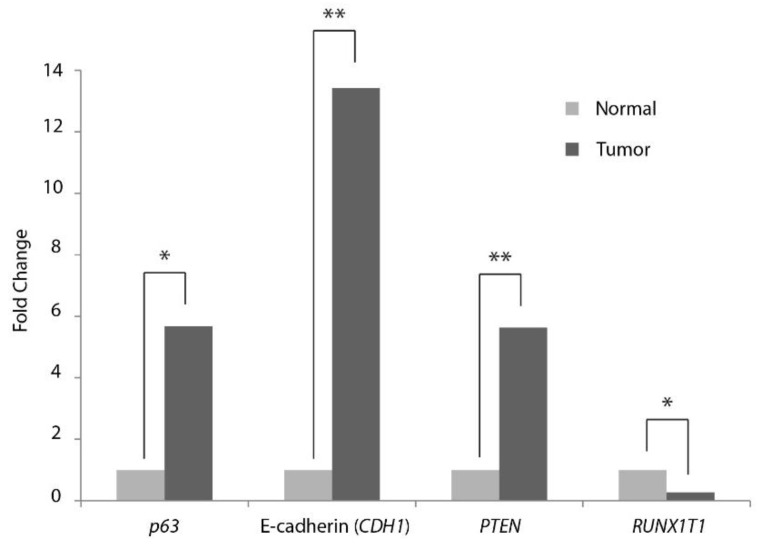
RT-qPCR re-evaluation of differentially expressed mRNA in meningioma. Fold changes of analyzed mRNA expression relative of 15 tumors, compared to four normal dura (3N +1NN) (see Table 1 and Table S5a). Three reference genes were used (*GAPDH*, *ACTB* (β-Actin) and *HPRT*). One asterisk (*) indicate *p*-value < 0.05 and two asterisk (**) indicate *p*-value < 0.001.

Ten putative mRNA targets, predicted by the identified differentially expressed miRNAs were selected for further analysis by RT-qPCR using 15 tumor samples and five controls (Table S4a). The mRNA expressions of *ALK7* (*ACVR1C*), *Cyclin G1* (*CCNG1*), *E2F5*, *mTOR*, *PTEN*, *RICTOR*, *RUNX1T1* (Cyclin D-related), *SIRT1*, *p53*, and *p63* were evaluated by RT-qPCR in meningioma and normal dura using *GAPDH* as a reference gene (Table S4a). This preliminary experiment did not show significant differences between Grade I and grade II, in these mRNAs (Table S4b). The experiment was repeated using three reference genes (*GAPDH*, *ACTB* and *HPRT*) and the five most promising targets mRNAs (*PTEN*, *p53*, *RICTOR*, *RUNX1T1* and *p63*), using 15 meningioma tumor samples and four dura controls N and NN (Table S5a). E-cadherin (*CDH1*) was included to assess and compare the expression in non-malignant meningiomas grades I and II with normal dura (N and NN). Validated mRNAs expression indicated that only three mRNA targets (*p63*, *PTEN* and *RUNX1T1*), in addition to E-cadherin, were significantly differentially expressed with fold changes larger than 3.7 (Figure 2; Table 4 and Table S5a). Grade I showed a significant difference *versus* grade II only in *RUNX1T1* (3.3 fold change) (Table S5b). mRNA expression shown in Figure 2, is given as ΔCq values with Standard deviation in Figure S2.

### 2.5. Immunohistochemical Examination

**Figure 3:**
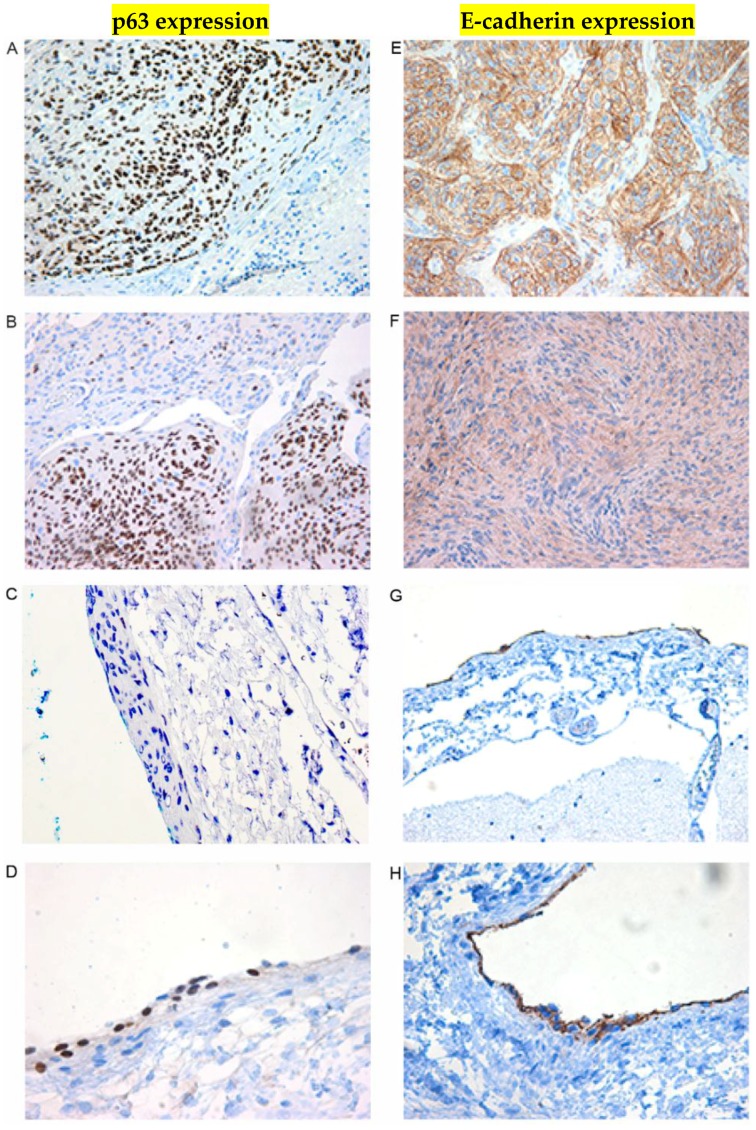
Immunohistochemistry of p63 (**A**–**D**) and E-cadherin (**E**–**H**) in meningioma (**A**, **B**, **E**, **F**) compared to normal arachnoid membranes (**C**, **D**, **G**, **H**). (**A**) Strong nuclear signal of p63 expression in this section of the tumor (200×); (**B**) Another tumor with both a moderate and a low signal for the nuclear p63 expression; (**C**) Immunohistochemistry p63 in arachnoid membrane from cadaver with no meningioma. No nuclear p63 expression in cells of the arachnoid membrane was observed in four different arachnoid autopsies as represented in C, at magnification of 400×; (**D**) Immunohistochemistry p63 in arachnoid from one sample with no meningioma a nuclear p63 expression in the external cap cells 400×; (**E**) Strong cytoplasmic signal of E-cadherin expression in this tumor (200×); (**F**) A tumor section with low E-cadherin expression; (**G**) Immunohistochemistry E-cadherin in arachnoid membrane from cadaver with no meningioma. Cytoplasmic E-cadherin expression is also limited to the outer layer of the arachnoid (200×); (**H**) Immunohistochemistry E-cadherin in arachnoid from cadaver with no meningioma in higher magnification (400×).

**Figure 4:**
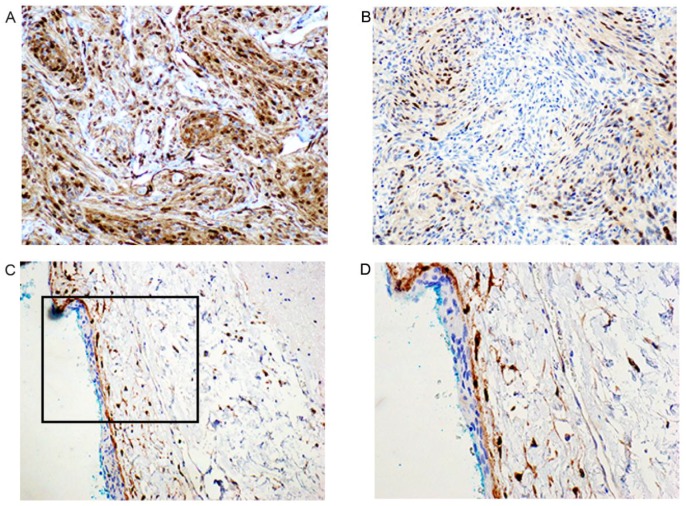
Immunohistochemistry of Cyclin D1 (**A**–**D**) in meningioma (**A**–**B**) compared to normal arachnoid membranes (**C**–**D**). (**A**) Meningioma with strong nuclear signal of Cyclin D1 expression (200×); (**B**) Another tumor with moderate signal for the nuclear Cyclin D1 expression (200×); (**C**) Immunohistochemistry Cyclin D1 in arachnoid membrane from cadaver with no meningioma. The external cap cell layer of the arachnoid membrane is negative, while some of the trabecular cells in the internal layer stain positive (200×); (**D**) The same as shown in C with higher magnification (400×).

IHC was included to further assess the increased expression of p63 in meningiomas grades I and II compared to normal arachnoid and dura membranes. Expression of p63 showed a various degree of density of nuclear distribution throughout the tumors (Figure 3A,B). Four of the normal arachnoid samples were negative for p63. However, one was positive for this marker in the external arachnoid cap cells (Figure 3C,D; Table 5). This high expression of cytoplasmic E-cadherin (13.4 fold; Figure 3, Table 4) was also observed by IHC (Figure 3E,F). In normal arachnoid E-cadherin expression was found in the external layer containing cap cells (Figure 3G,H; Table 5). Furthermore, IHC was used to detect the expression of Cyclin D1 (Figure 4, Table 5). Nuclear expression of Cyclin D1 was present as a strong or moderate signal throughout the meningiomas regardless of being grades I or II. In four of the normal arachnoid autopsies, no signal for Cyclin D1 could be detected in the external cap cells, but some of the trabecular cells in the internal layer stained positive (Figure 4, Table 5). In one arachnoid sample, we found positivity related to the external cap cells, noting that this was the same sample that stained positive for p63 in Figure 3D.

## 3. Discussion

### 3.1. Differentially Expressed Micro RNAs

Deep sequencing analysis of small RNAs of meningioma grades I and II revealed differentially expressed miRNAs when comparing meningioma (grade I or grade II) *versus* the normal dura (N and NN) as well as grade I *versus* grade II. After verification of the top 18 miRNAs by RT-qPCR with fold change values over ±5.17 in any of these comparisons, only six miRNAs (miR-21, miR-34a, miR-143, miR-193b, miR-218, and miR-451) were clearly differentially expressed (tumors *vs.* normal). The down-regulation of miR-21 and miR-34a seen in SOLiD sequencing data between grades I and II was not verified by RT-qPCR (Table S3b). However, a significant fold change of −3.7 of miR-21 and 3.133 of miR-34a in tumors relative to the normal control using tumors from 15 patients and five normal dura controls (Table S3a) necessitated their inclusion in the discussion. It should be noted that the differential expression between grades I and II observed in deep sequencing in a few miRNAs (miR-21, miR-34a, miR-376, miR-451 and miR-99a) were not verified in RT-qPCR (Table 2 and Table 3). This could be due to the small number of sequenced samples.

There are several recent reports on the expression of miRNA in meningioma grades I, II, and III. None of these studies are based on deep sequencing, but they depend on pre-selected miRNA microarray or RT-qPCR array approaches. Interestingly, the list of the 40 most differentially expressed meningioma miRNAs by Saydam *et al.* [43], is different from that of our dataset. In another study [44], a similar feature was seen. These limited overlaps between meningioma miRNA profiles in three different studies might suggest that meningiomas are diverse in their gene regulation mechanisms. However, it is also possible that these discrepancies are due to variations in the technical performance between different profiling platforms [45,46].

We observed a down-regulation −4 fold, (RT-qPCR) of the oncogenic miR-21 in meningioma relative to normal dura tissue. This finding differentiates non-malignant meningioma from cancers and anaplastic meningiomas. It was reported that miR-21 is upregulated in most cancers [47], as well as in meningioma grade III [48] and in Glioblastoma multiforme [49]. Two tumor suppressor miRNAs were found to be significantly downregulated in the studied tumor samples. miR-143 (−5/−4 fold, SOLiD/RT-qPCR) has been reported to be associated with tumor size and metastasis in cervical squamous cell carcinoma [50], as well as poor prognosis in endometrioid carcinoma due to increased expression of DNA methyltransferase 3B [51]. miR-193b (−6/−4 fold, SOLiD/RT-qPCR) down-regulation has been observed in cancer [52,53] suggesting that its pro-proliferation mRNA targets are not inhibited in cancers as well as in meningiomas. Interestingly, a high abundance of miR-218 (+8/+4 fold, SOLiD/RT-qPCR) was observed in the studied meningiomas. miR-218 is considered a tumor suppressor [54,55], suggesting that miR-218 also play a role in preventing or slowing meningioma grades I and II from malignant progression. We observed strong down-regulation (−10/−18 fold, SOLiD/RT-qPCR) of miR-451 in meningiomas. miR-451 is considered a tumor suppressor that inhibits proliferation and invasion by regulating epithelial-to-mesenchymal transition (EMT) in bladder cancer [56] and hepatocellular carcinoma [57]. The down-regulation of miR-451 suggests that its pro-proliferation mRNA targets may not be inhibited in meningioma grades I and II. Finally, in the validation experiments with RT-qPCR, the potent tumor suppressor miR-34a was found to be overexpressed (3.1 fold) in all studied meningiomas. miR-34a contributes to p53 downstream effects on proliferation arrest and induction of apoptosis, by targeting c-MYC, c-MET and a long list of genes involved in different oncogenic processes, including inhibiting EMT [58]. The differential expression of miR-218, miR-34a and miR-451 in meningiomas were previously reported, using microarray analysis [48]. Also, miR-451 differential expression was reported in meningioma by microarray analysis in another study [43].

### 3.2. Differentially Expressed mRNA Selected Putative Targets

Among the ten selected putative mRNA targets for the six differentially expressed miRNAs, the expression of *p63* was found to be upregulated (+6 fold, RT-qPCR) in meningioma. p63 expression, as evaluated by IHC, was found to be nuclear and throughout the tumors. The cap cells of the normal arachnoid were negative for p63 as observed in four autopsies. In one sample, p63 expression was detected in the cap cells. This arachnoid sample also had some Cyclin D1 positive cap cells, but it is not clear if this deceased patient was developing meningioma. Mittal and coworkers [59] demonstrated by IHC positive nuclear p63 staining in meningioma that it increased with higher grades (II and III). More arachnoid autopsies should be analyzed in order to elaborate on the significance of p63 expression in the cap cells, as well as its potential significance in meningioma.

The observed higher expression of the E-cadherin (+13 fold, RT-qPCR) throughout the studied meningioma tumors might also contribute to the non-anaplastic nature. E-cadherin is encoded by the tumor suppressor gene *CDH1* [60], and represents the core protein of the epithelial adherens junction. Through its cytoplasmic domain it interacts with several signaling proteins [61]. Reduced E-cadherin expression is associated with short overall survival in cancer [62,63]. Reduced E-cadherin expression was also observed in anaplastic meningiomas (grade III) [3,64,65]. In normal arachnoid tissues, the expression of E-cadherin was observed only in the outer cap-cell layer. This could be one of the distinguishing features of the arachnoid, contributing to the function of these tightly packed arachnoid cap cells. The observed over-expression of E-cadherin (*CDH1*) in the current study represents an additional factor in favor of preventing malignant progression in meningiomas grades I and II.

Another significant finding was that the *PTEN* is overexpressed (+6 fold, RT-qPCR) compared to that of normal meninges controls (N and NN tissues). *PTEN* expression supports the non-malignant nature of meningioma grades I and II. In a previous study of *PTEN*, the histoscore was reported to be inversely correlated with recurrence probability in meningioma [66]. The observed overexpression of *PTEN* in meningioma is probably linked to the downregulation of miR-21, since *PTEN* is a target for miR-21 [67]. The expression of *PTEN* and E-cadherin might act synergistically to contribute to the non-malignant nature of meningioma grades I and II. E-cadherin has been reported to affect the expression of *PTEN* [68]. Why *PTEN* is overexpressed in meningioma relative to meninges (dura, N and NN), and if it is related to a higher level of E-cadherin, are not currently known. However, the *PTEN* promoter has been found to be hypomethylated in meningioma grades I and II [69], suggesting the contribution of epigenetic regulation.

The tumor suppressor *RUNX1T1* expression was lower in meningiomas grades I and II compared to that of the controls (−4 fold, RT-qPCR). A significant fold change of 3.3 is shown between grade I *versus* grade II when three reference genes were used (Table S7b), which also supports its anti-proliferation function. miR-193b presumably targets RUNX1T1 mRNA, however in this study miR-193b was also downregulated (Table 3). It is likely that other miRNAs that can target RUNX1T1 are expressed in meningioma and could be involved. Other epigenetic factors could also be involved. RUNX1T1 is a member of RUNX proteins that belong to a family of metazoan transcription factors [70]. The RUNX protein family serves as master regulators of development, and is frequently deregulated in human cancers [70].

Cyclin D1 expression assessed by IHC revealed a strong or moderate expression throughout all studied meningiomas regardless of being grades I or II. In normal arachnoid tissue Cyclin D1 was confined to a few cells in the sub-arachnoidal space, and was generally not observed in the external cap cells. Cyclin D1 belongs to the core cell cycle machinery and its overexpression is frequently associated with cancer [71]. In a recent study, all grades of meningiomas exhibited Cyclin D1 expression, and Cyclin D1 level which increased at higher grades was linked to poorer prognosis [72].

## 4. Materials and Methods

### 4.1. Ethical Considerations

All meningiomas and dura controls were obtained from patients after approval from the Regional Ethical Committee (REC) (REK Nord ref No. 2010/1619) at the University Hospital of North Norway, Tromsø. Informed written consent was obtained from all the participants. The arachnoid samples were collected from autopsy specimens submitted for neuropathological examination.

### 4.2. Patient Samples

All samples (tumors and controls) used in this study are listed in Table 1. The histological classification of meningiomas followed the WHO grading system (Table S1a) according to [3]. Subsequently, meningiomas grade I were further classified into different morphological subgroups (A to H), based on their histology (Table S1b). This morphological sub-classification for grade I is referred to in all subsequent tables, for example, for subclass A as IA, for sub class B as IB *etc.* During surgery, fresh meningioma tissues (grades I and II) were removed from the tumors of 15 patients and small samples were directly frozen in liquid nitrogen. In patients 1 and 2, in addition to four other patients, tissues from normal dura (N) well outside the tumors were also collected and directly frozen in liquid nitrogen. Three dura controls were harvested from patients without tumors (NN) during subdural hematoma surgery.

For the IHC supporting studies, arachnoid tissues were used as controls (NN-a). These arachnoid tissues were obtained from cadavers without meningioma. The biopsies of dura were used as the normal control for sequencing studies. All samples were frozen and kept in liquid nitrogen until RNA isolation. Isolated RNA samples were kept at −80 °C until further use.

Tumors from two patients were subjected to small RNA deep sequencing. Patient 1; grade IA tumor (Table 1) was a 71 year-old man admitted due to limb ataxia. Examinations disclosed lack of smelling function (anosmia) and the MRI showed a tumor (meningioma) attached to the skull floor between the two frontal cerebral lobes (Figure 1A). The tumor was removed without any further neurological deficits. Patient 2; grade II tumor (Table 1) was a 74 year-old man that suffered from progressive hemiparesis and dementia over the last few years. The tumor (Figure 1B) located in the motor region was removed completely without any further neurological deficits.

### 4.3. Immunohistochemistry (IHC)

Staining for Ki-67, p63, E-cadherin and Cyclin D1 were all performed using the Ventana BenchMark XT/ULTRA automated slide preparation system (Ventana Medical Systems, Inc., Tucson, AZ, USA) according to the instructions from the manufacturer. The primary antibodies were all from Roche, monoclonal anti-rabbit for Ki-67, E-Cadherin (EP700U) and Cyclin D1 (SP4-R), monoclonal anti-mouse for p63 (p63-4A4; against all the six forms of p63).

### 4.4. Total RNA Extraction

Total RNAs were isolated from all tumors and controls (Table 1). Tumor biopsies were homogenized in Trizol (Life Technologies Corporation, Carlsbad, CA, USA) by bead milling with ceramic beads (Roche Applied Science, Basel, Switzerland). The dura control biopsies were homogenized in liquid nitrogen prior to RNA isolation in Trizol. All RNA isolations included prolonged precipitation and centrifugation steps in order to preserve the small RNA fractions. Phase separation was made by incubation on ice for 30 min followed by centrifugation at 12,000 g for 20 min at 4 °C. Total RNA samples were precipitated overnight on ice and centrifuged at full speed (21,000 g) for 30 min at 4 °C. Concentration and quality of RNA was measured with Quant-IT RNA assay kit (Life Technologies Corporation) with Qubit Fluorimeter (Life Technologies Corporation) and Agilent RNA 6000 Nano kit with Agilent 2100 Bioanalyzer (Agilent Technologies Inc., Santa Clara, CA, USA), respectively. RIN values for all isolated RNA samples are listed in Table S6.

### 4.5. SOLiD Sequencing

Total RNA of meningioma tumor and dura samples (N) from patient 1 (grade I) and patient 2 (grade II), in addition to dura controls form one patient without meningioma (NN) (Table 1; Table S1a,b) were subjected to SOLiD sequencing as described in the SOLiD Small RNA Expression Kit Protocol (Life Technologies Corporation). FlashPAGE™ Fractionator (Life Technologies Corporation) enriched total RNA samples for small RNA species. Approximately 50 ng of enriched samples were subjected to adaptor ligation and subsequently to reverse transcription and RNase H digestion. The cDNAs were amplified using barcoded primers and the desired PCR products were purified by size selection (105 nt to 150 nt) on 6% PAGE. Equal molar amounts of each barcoded sample were pooled together in one library and used as a template in emulsion PCR (Life Technologies Corporation) followed by SOLiD-4 sequencing (Life Technologies Corporation). SOLiD sequencing was performed at the genomic facility, Nord University, Bodø, Norway.

### 4.6. Sequence Data Analysis

Approximately 74, 84 and 75 million raw sequence reads of small RNAs were obtained from one normal patient without meningioma (NN), from normal dura and meningioma from patient 1, and from normal dura and meningioma from patient 2, respectively. The obtained raw color-space data was analyzed using CLC Genomics Workbench (CLCbio, Aarhus, Denmark). Adaptors were trimmed and sequences were grouped and counted. All tags with less than 50 reads were removed from the dataset. The remaining tags were annotated against known human miRNAs of MirBase v17 [15]. Successful annotation of a miRNA was stringent and did not include substitutions or length heteroplasmy. Annotated miRNA reads counted 9,565,805 (Normal), 12,185,832 (IA-grade), and 1,304,790 (II-grade). For comparison purposes, the reads were normalized using linear total count scaling. In fold change studies, an additional cut-off step of 500 reads/million was introduced on normalized reads. Table S2a,b include a summary of these data.

### 4.7. Validation of miRNA Expression in Meningioma and Normal Dura by Real-Time Quantitative PCR (RT-qPCR)

The relative expression of miRNAs was analyzed by RT-qPCR. Total RNA of all tumors and dura biopsy controls were analyzed by miRCURY LNA™ Universal RT microRNA PCR Pick-&-Mix panels (Exiqon A/S, 2950 Vedbaek, Denmark). Target mRNA and primer catalog numbers are listed in Table S7. Included in the panels were three Inter-Plate Calibrators (IPC) and three candidate miRNA reference genes (hsa-miR-191, hsa-miR-16 and hsa-let-7a) [42]. Following the manufacturer’s instructions, the amplification was run on the Applied Biosystems 7900HT instrument. Raw Cq values were calculated by the SDS software v2.4 (Life Technologies Corporation) with automatic baseline setting and manual ΔRn threshold of 2.5 for all assays. Cq values were adjusted by IPC and normalized using hsa-miR-191, hsa-miR-16 and hsa-let-7a as indigenous reference miRNA genes, in accordance with the ΔΔCq method. Fold change analysis was performed using Microsoft^©^ Excel, 2010 version 14 Microsoft Corp, Redmond, WA, USA). Student’s t-test was used for calculating *p*-values.

### 4.8. Validation of Selected Possible mRNA Targets Expression in Benign Meningiomas and Normal Dura Biopsies (N and NN) by RT-qPCR

Total RNA samples were DNase-treated with a Heat and Run gDNA removal kit (ArcticZymes, Tromsø, Norway) prior to cDNA synthesis with an iScript kit (Bio-Rad, Hercules, California, CA, USA), both according to the manufacturer’s protocol. The samples were profiled for the relative expression of 10 selected mRNA targets and subsequently E-cadherin was added to the list, using the TaqMan system (Life Technologies Corporation), specific primers and assay ID (listed in Table S8). The three reference genes used are *GAPDH*, *ACTB* (β-Actin) and *HPRT.* All calculations were performed as described above for the validation of miRNA expression, except that manual ΔRn threshold was set to 0.2.

## 5. Conclusions

Three out of the eleven differentially expressed biomarkers are reported for the first time in meningioma (Table 6). Our results indicate that meningioma tumor formation and proliferation in part could be attributed to the lower expression of miR-143, miR-193b and miR-451, a feature similar to that of several malignant tumors. Low expression of the tumor suppressor *RUNX1T1,* in concert with the expression of *p63* and Cyclin D1 could be contributing factors to the tumor growth in the non-anaplastic meningiomas. Overexpression of *p63,* which is considered as a pro-proliferation gene, may also contribute to tumor growth. Furthermore, our data indicate that the expression of E-cadherin (*CDH1*) appeared as a good prognostic marker in these intracranial non-anaplastic tumors. This notion is supported by the fact that its expression level is reduced in anaplastic meningioma. The down-regulation miR-21, combined with the overexpression of miR-34a, miR218, *PTEN* and E-cadherin (*CDH1*) could explain the benign nature of meningiomas (grades I and II) and represent barriers for grades I and II tumors from malignant progression.

## Figures and Tables

**Table 1:** List of meningiomas used for RNA isolation for SOLiD small RNA sequencing and for the validation of miRNAs and mRNA expression studies by RT-qPCR, as well as IHC analysis.

Patient No.	Sample Type	Histology Grade/Subclass *	Gender	Age	Notes
1	T + N	IA	M	71	+ Deep sequencing
2	T + N	II	M	74	+ Deep sequencing
3	T	II	M	60	
4	T	IA + D	F	70	
5	T	IB	F	68	
6	T	II	M	75	
7	T	IA	M	69	
8	T	IC	F	55	
9	T + N ***	IC	F	42	
10	T + N	IA	M	68	
11	T	IC	F	62	
12	T	IC	M	47	
13	T + N	IA	F	35	
14	T ***	IC	F	43	
15	T + N	IC	M	59	
16	NN	Normal	M	80	+ Deep sequencing
17 (cadaver)	NN-a	Arachnoid	M	55	
18 (cadaver)	NN-a	Arachnoid	M	59	
19 (cadaver)	NN-a	Arachnoid	M	73	
20 (cadaver)	NN-a	Arachnoid	M	49	
21 (cadaver)	NN-a	Arachnoid	M	54	
22	NN	Normal	M	64	
23	NN **	Normal	M	76	Only Deep sequencing

*: WHO grades I and II according to criteria given in Table S1a; Subgroup according to letters given in Table S1a and S1b. T refers to tumor sample, N refers to dura control from patient with tumor, NN refers to dura control from patient without tumors. NN-a samples are arachnoid controls from cadavers. **: This patient NN Dura was used for SOLiD sequencing, but not for RT-qPCR analysis. ***: The N-sample from patient nine and the T-sample from patient 14 were manually removed from the mRNA analysis due to technical variations.

**Table 2:** Differentially expressed miRNAs based on SOLiD sequencing data. The selection of putative mRNA targets is based on publications in the field of cancer and cell proliferation, not on target prediction tools.

miRNA	Change Related to	* Fold Change	Selected 10 Putative Target mRNA for Analysis by RT-qPCR
let-miR-7g	II/N	+10.48	*Cyclin D*, *E2F*, *p53* [24]
miR-122^1^	II/N	−81.56	*Cyclin G1* [25]
miR-17	II/N	−10.34	*Cyclin D1*, E2F1, PTEN [26]
miR-130a	II/N	+6.32	
miR-143	IA/N	−5.17	
miR-148b	II/N	−6.02	
miR-152	II/N	+6.31	*E2F3, RICTOR* [27]
miR-193b	IA/N	−5.76	*RUNX1T1* (*Cyclin D*-related) [28], *(Cyclin D1)* [29]
miR-199a-5p	IA/N	−6.20	E2F3 [30]
miR-21	IA/II:	−5.45	*PTEN* [31]
miR-218	II/N	+8.55	*RICTOR* [32,33]*, mTOR* [32], regulating p53 [34]
	IA/N	+7.59	
miR-26b	IA/II	+8.86	Activation of *PTEN–Akt* pathway [35]
miR-34a	IA/II	−8.51	*Sirt1* [36]
miR-342-3p	IA/N	+7.52	
miR-376c	II/N	+8.14	*ALK7* [37]
	IA/II	−7.92	
miR-424	II/N	+6.33	
miR-451	IA/N	−10.07	*PI3K/Akt/mTOR* signaling pathway [38]
	IA/II	−6.90	
miR-574-3p	IA/II	+13.12	*P63* [39]
miR-99a	IA/II	−6.09	*mTOR* [40,41]
	II/N	+5.42	

^1^: This large drop of expression was not found in any other tumor sample in a preliminary RT-qPCR and was not included in further validation experiments. *: Fold change ±5.17 folds, selected for validation by RT-qPCR.

**Table 3:** Overview of the RT-qPCR validation of miRNA expression with fold change and p-value, average tumor *vs.* average normal dura. Summary of significant (*p*-value <0.05) miRNAs with fold change above three are marked in bold. Human miR-191, miR-16 and let-7a are reference miRNA genes [42]. 15 tumor samples were used (12 different grade I tumors, and three grade II tumors) in comparison to five normal dura controls (see Table S3a).

miRNA	Fold Change	*p*-Value
let-7g	1.132	0.444
miR-130a	2.254	0.004
**miR-143**	**−3.867**	**0.001**
miR-148b	1.522	0.041
miR-152	2.218	0.000
miR-17	−2.089	0.000
**miR-193b**	**−4.063**	**0.007**
miR-199a-5p	−1.548	0.325
**miR-21**	**−3.710**	**0.003**
**miR-218**	**4.473**	**0.003**
miR-26b	1.156	0.377
miR-342-3p	1.146	0.496
**miR-34a**	**3.133**	**0.002**
miR-376c	1.372	0.395
miR-424	1.806	0.103
**miR-451**	**−18.452**	**0.000**
miR-574-3p	−1.286	0.212
miR-99a	1.110	0.760

**Table 4:** RT-qPCR analysis of mRNA expression, average tumor *vs.* average normal dura. mRNAs with significant *p*-values (<0.05) are in bold. Three reference genes were used (*GAPDH*, *ACTB* (β-Actin) and *HPRT*).

mRNA	Fold Change	*p*-Value
***p63***	5.7	0.044
**E-Cadherin (*CDH1*)**	13.4	0.000
***PTEN***	5.6	0.000
***RUNX1T1* (Cyclin D related)**	−3.7	0.021
*RICTOR*	1.5	0.298
*p53*	1.5	0.213

**Table 5:** Immunohistochemistry of p63, E-Cadherin and Cyclin D1 of meningioma (grades I and II), and biopsies of dura and arachnoid autopsies of non-meningioma patients. See Table 1.

Patient NO.	Sample Type	Histology	P 63 (Nucleus)	E-Cad (Membrane)	Cyclin D1 (Nucleus)
1	T	IA	++	++(+)	++
2	T	II	+	++	++(+)
3	T	II	+	++(+)	+
4	T	IA+IB	++	++(+)	+
5	T	IB	-	+	++(+)
6	T	II	+++	+++	+++
7	T	IA	+	++(+)	+++
8	T	IC	+	++	++
9	T	IC	++	++	++
10	T	IA+II	+	+++	+++
11	T	IC	++	++	+++
12	T	IC	++	++	++(+)
13	T	IA	++(+)	+++	+
14	T	IC	+	+++	++(+)
15	T	IC	++	++	++(+)
					
17	Arachnoidea Autopsy	Normal (NN-a)	+	+	+
18	Arachnoidea Autopsy	Normal (NN-a)	-	+	Negative in cap cells. Positive in some of the trabecular cells in the subarachnoidal space
19	Arachnoidea Autopsy	Normal (NN-a)	-	+	As previous
20	Arachnoidea Autopsy	Normal (NN-a)	-	+	As previous
21	Arachnoidea Autopsy	Normal (NN-a)	-	+	-
22	Dura	Normal NN	-	+	Positive in single cells, not well oriented

For tumor samples: - : no cells positive; +: focal positivity; ++: positivity in close to 50% of tumor cells; ++ (+): Positivity in most cells, but areas with weak staining; +++: Strong positivity in over 50% of tumor cells; For arachnoidea and dura controls: +: indicates positive cap cells; -: indicates negative cap cells.

**Table 6:** Summary of putative pro- and anti-proliferation differentially expressed miRNAs and mRNAs/proteins in meningioma.

For Proliferation	^n^ Code	Expression Relative to Controls	Prognosis	Anti-Proliferation/Anti-Malignancy	^n^ Code	Expression Relative to Controls	^nn^ Prognosis
miR-143 ^1st^	TS	Under	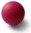	miR-21	ONC	Under	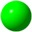
miR-193b ^1st^	TS	Under	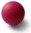	miR-34a	TS	Over	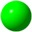
miR-451	TS	Under	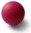	miR-218	TS	Over	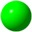
*RUNX1T1* ^1st^	TS	Under	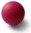	*PTEN*	TS	Over	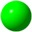
*P63*; also positive by IHC	Pro-Growth	Over	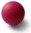	E-cadherin (*CDH1*); also positive by IHC	TS	Over	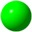
Cyclin D1	ONC	Over; by IHC	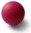				

^n^: Code for tumor suppressor is TS and for oncogene, ONC; ^nn^: Non-malignant appeared to be the dominant phenotype; ^1st^: Reported in meningioma for the first time. Also, its expression in grade II was lower than grade I. 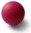 Bad prognostic sign, 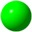 Good prognostic sign.

## Supplementary Material

Supplemental material can be accessed at: http://www.mdpi.com/2072-6694/8/3/31/s1. **Table S1a**: World Health Organization (WHO) grading of meningiomas. CBTRUS; Central Brain Tumor Registry of the United States http://www.cbtrus.org/2011-NPCR-SEER/WEB-0407-Report-3-3-2011.pdf. **Table S1b**: Sub-classification of meningioma. **Table S2a**: MicroRNA sequence analysis and fold change. **Table S2b**: Summary of normalized (by totals) SOLiD miRNA reads, values in reads pr. million for total miRNAs from meningioma tumors (T) or dura (N or NN) (from patients (P)/Samples; as in Table 1). **Table S2c**: Global comparisons of all expressed miRNAs between grades I and II, as well as between N and NN controls. **Table S3a**: miRNA RT-qPCR analysis. The ΔΔCq method was used to calculate all the fold changes and the Student’s t-test (two tailed) was used to calculate all the p-values. **Table S3b**: Relative expression of microRNAs of grade I *versus* grade II for the miRs that exhibited differential expression between tumors *versus* controls as shown in Table 3. Fold change between grades I and II and the p-values are shown. The ΔΔCq method was used to calculate all the fold changes and the Student's t-test (two tailed) was used to calculate all the *p*-values. **Table S4a**: Initial RT-qPCR mRNA data analysis of meningiomas compared to control. The ΔΔCq method was used to calculate all the fold changes and the Student's t-test (two tailed) was used to calculate all the *p*-values. **Table S4b**: Initial RT-qPCR mRNA data analysis of meningiomas grade I compared to grade II did not reveal a significant differences in the tested mRNAs using one reference gene. **Table S5a**: RT-qPCR re-evaluation of 6 mRNA differential expression (tumors *versus* control) with three reference genes. The ΔΔCq method was used to calculate all the fold changes and the Student's t-test (two tailed) was used to calculate all the *p*-values. **Table S5b**: Relative expression of mRNA of grade I *versus* grade II for the mRNAs using RT-qPCR for mRNA presented in Table 4. Fold change between grades I and II and the p-values are shown. The ΔΔCq method was used to calculate all the fold changes and the Student’s t-test (two tailed) was used to calculate all the p-values. **Table S6**: RIN values for RNA isolated from tumors and controls (N and NN). **Table S7**: RT-qPCR re-evaluation-primers for miRNA expression (Exiqon A/S, 2950 Vedbaek, Denmark). **Table S8**: Assay ID for selected putative mRNA targets (Life Technologies Corporation, Carlsbad, CA, USA). **Figure S1**: miRNA expression in meningioma relative to normal control. Expression values are given as ΔCq values, hence lower ΔCq values are equal to higher expression. Standard deviation is shown as vertical lines. **Figure S2**: mRNA expression in meningioma relative to normal control. Expression values are given as ΔCq values, hence lower ΔCq values are equal to higher expression. Standard deviation is shown as vertical lines.
