# Supplementary material for: Differentially Expressed MicroRNAs in Meningiomas Grades I and II Suggest Shared Biomarkers with Malignant Tumors
Source: Cancers (Basel). 2016 Mar 3;8(3):31. doi: 10.3390/cancers8030031 (PMC4810115; doi:10.3390/cancers8030031)

Supplementary Materials

Table S1a. World Health Organization (WHO) grading of meningiomas. CBTRUS; Central Brain Tumor Registry of the United States http://www.cbtrus.org/2011-NPCR-SEER/WEB-0407-Report-3-3-2011.pdf.

WHO Grade	Description
**I**	Meningiomas without features of grade II or III lesions. Low risk of recurrence and/or aggressive growth.
**II**	Atypical meningiomas, with brain invasion, and/or increased mitotic activity (defined as four or more per 10 high-power field (HPF),- though fewer than 20 mitoses per 10 HPF) and/or three or more of the following features: increased cellularity, small cells with high nucleus-to-cytoplasm ratio, prominent nucleoli, uninterrupted pattern-less or sheet-like growth, and foci of spontaneous or geographic necrosis.<br>Chordoid meningioma and Clear cell meningioma are also classified Grade II.
**III**	Anaplastic (malignant) meningiomas exhibit histological features of malignancy far in excess of the abnormalities present in atypical meningiomas. These features are increased mitotic rate (20 mitosis or more per 10 HPF) or malignant cytology like the morphology found in carcinoma, melanoma or high-grade sarcoma.<br>Rhabdoid meningioma and papillary meningioma are also classified Grade III.

In addition to the WHO grading the tumors are sub classified according to their morphological diversity, see Table S1b.

Table S1b. Sub classification of meningioma**.**

Letter used in this study	Cell morphology
A	Meningothelial
B	Fibrous
C	Transitional (mixed A and B)
D	Psammomatous
E	Angiomatous
F	Microcystic
G	Sectretory
H	Lymphoplasmocyte-rich

Table S2a. MicroRNA sequence analysis and fold change. P, patient (numbers and samples as in Table 1).

Menigioma project

SOLiD
Next Generation Sequencing

microRNA profile

Name	P2-N vs P2-T	P1-N vs P1-T	P2-N vs P16-NN	P1-N vs P16-NN	P2-N vs P1-N	P2-T vs P1-T	Name
hsa-let-7a	1,03	1,28	-1,29	-1,13	-1,14	1,09	hsa-let-7a
hsa-let-7b	-2,92	-2,33	-1,43	-1,10	-1,29	-1,03	hsa-let-7b
hsa-let-7b_star	DOWN (1289)		DOWN (1289)		DOWN (1289)		hsa-let-7b_star
hsa-let-7c	-1,08	-2,54	-2,11	-1,59	-1,33	-3,11	hsa-let-7c
hsa-let-7d	1,41	2,19	2,49	1,81	1,38	2,14	hsa-let-7d
hsa-let-7e	4,87	1,41	2,03	1,03	1,98	-1,74	hsa-let-7e
hsa-let-7f	2,56	1,92	1,39	-1,02	1,42	1,07	hsa-let-7f
hsa-let-7g	10,48	1,40	3,03	-1,04	3,14	-2,39	hsa-let-7g
hsa-let-7i	-1,16	DOWN (1167)	DOWN (1052)	DOWN (1167)	1,11	DOWN (910)	hsa-let-7i
hsa-miR-100	1,69	1,64	-1,29	-1,84	1,43	1,38	hsa-miR-100
hsa-miR-101	6,54	-1,40	2,07	-1,80	3,74	-2,45	hsa-miR-101
hsa-miR-103a	3,98	2,58	4,05	1,88	2,15	1,40	hsa-miR-103a
hsa-miR-106b		-1,17	UP (768)	1,25	UP (613)	UP (525)	hsa-miR-106b
hsa-miR-107	4,57	1,91	1,78	1,30	1,36	-1,75	hsa-miR-107
hsa-miR-10a	DOWN (1205)		DOWN (1205)		DOWN (1205)		hsa-miR-10a
hsa-miR-10b	DOWN (1088)	DOWN (1576)	1,47	1,02	1,45		hsa-miR-10b
hsa-miR-122	-81,56		DOWN (283989)		DOWN (283989)	DOWN (3482)	hsa-miR-122
hsa-miR-122_star	DOWN (2401)		DOWN (2401)		DOWN (2401)		hsa-miR-122_star
hsa-miR-125a-5p	2,29	2,35	2,15	-1,07	2,30	2,36	hsa-miR-125a-5p
hsa-miR-125b	2,34	2,30	1,84	1,05	1,75	1,73	hsa-miR-125b
hsa-miR-126	-1,86	-3,78	3,43	-1,63	5,59	2,75	hsa-miR-126
hsa-miR-126_star	-1,61	-5,17	3,30	-1,12	3,68	1,14	hsa-miR-126_star
hsa-miR-1260	1,06	UP (698)	-1,37	UP (1295)	DOWN (1774)	-2,68	hsa-miR-1260
hsa-miR-1260b	-1,33	UP (830)	-1,66	UP (1566)	DOWN (2601)	-2,36	hsa-miR-1260b
hsa-miR-128	DOWN (2520)	UP (664)	-4,46	UP (565)	DOWN (2520)	UP (664)	hsa-miR-128
hsa-miR-130a	6,32	1,02	3,01	-2,16	6,49	1,04	hsa-miR-130a
hsa-miR-130b			UP (576)	UP (576)			hsa-miR-130b
hsa-miR-134			UP (581)	UP (581)			hsa-miR-134
hsa-miR-136	UP (1500)					DOWN (1500)	hsa-miR-136
hsa-miR-138	DOWN (839)	UP (1400)	DOWN (839)		DOWN (839)	UP (1400)	hsa-miR-138
hsa-miR-139-5p	DOWN (893)		1,82	UP (1626)	DOWN (893)		hsa-miR-139-5p
hsa-miR-140-3p	-1,02	-2,82	2,66	-2,44	6,50	2,34	hsa-miR-140-3p
hsa-miR-140-5p	1,55	-4,19	1,59	-3,20	5,09	-1,28	hsa-miR-140-5p
hsa-miR-142-3p	1,31	DOWN (1118)	1,44	-1,12	1,62	DOWN (905)	hsa-miR-142-3p
hsa-miR-142-5p	DOWN (974)		DOWN (974)		DOWN (974)		hsa-miR-142-5p
hsa-miR-143	-1,74	-5,17	1,24	-1,59	1,98	-1,50	hsa-miR-143
hsa-miR-144	1,28	DOWN (3042)	DOWN (1310)	DOWN (3042)	2,32	DOWN (1673)	hsa-miR-144
hsa-miR-144_star	DOWN (2733)	DOWN (796)	DOWN (2733)	DOWN (796)	-3,43		hsa-miR-144_star
hsa-miR-145	-2,46	-2,11	2,03	1,50	1,35	1,57	hsa-miR-145
hsa-miR-146a	UP (998)	DOWN (621)		DOWN (621)	UP (621)	DOWN (998)	hsa-miR-146a
hsa-miR-148a	2,52	2,77	1,00	1,19	-1,19	-1,08	hsa-miR-148a
hsa-miR-148b	-6,02		DOWN (6380)		DOWN (6380)	DOWN (1061)	hsa-miR-148b
hsa-miR-149	DOWN (587)		DOWN (587)		DOWN (587)		hsa-miR-149
hsa-miR-150	4,58	-2,49	6,94	3,31	2,10	-5,45	hsa-miR-150
hsa-miR-151-3p		-1,23	UP (620)	-2,05	UP (1271)	UP (1034)	hsa-miR-151-3p
hsa-miR-151-5p	3,10	1,43	5,34	-1,13	6,06	2,79	hsa-miR-151-5p
hsa-miR-152	6,31	1,13	1,80	-1,16	2,09	-2,66	hsa-miR-152
hsa-miR-15a	-4,15	-1,08	-1,20	1,61	-1,93	1,99	hsa-miR-15a
hsa-miR-15b	-2,37	-1,30	2,21	3,16	-1,43	1,28	hsa-miR-15b
hsa-miR-15b_star	DOWN (818)		DOWN (818)		DOWN (818)		hsa-miR-15b_star
hsa-miR-16	DOWN (1807)	-1,42	1,99	1,78	1,12	UP (1430)	hsa-miR-16
hsa-miR-16-2_star	DOWN (1627)	DOWN (865)	DOWN (1627)	DOWN (865)	-1,88		hsa-miR-16-2_star
hsa-miR-17	-10,34	-1,39	-9,93	-1,82	-5,46	1,36	hsa-miR-17
hsa-miR-181a	3,08	3,31	9,45	5,90	1,60	1,72	hsa-miR-181a
hsa-miR-181b	1,31	1,60	2,31	2,46	-1,06	1,15	hsa-miR-181b
hsa-miR-181c		UP (792)				UP (792)	hsa-miR-181c
hsa-miR-185	-2,54	-1,88	-1,46	1,15	-1,68	-1,25	hsa-miR-185
hsa-miR-186	1,11	-1,97	2,79	-1,65	4,60	2,10	hsa-miR-186
hsa-miR-191	1,44	2,66	2,27	1,21	1,87	3,45	hsa-miR-191
hsa-miR-192	DOWN (539)	1,71	2,01	-2,16	4,35	UP (4020)	hsa-miR-192
hsa-miR-193a-5p			UP (864)	UP (864)			hsa-miR-193a-5p
hsa-miR-193b	DOWN (1304)	-5,78	2,51	1,10	2,28	UP (514)	hsa-miR-193b
hsa-miR-195	UP (781)	-3,19	UP (4378)	-1,37	UP (5993)	2,40	hsa-miR-195
hsa-miR-197	-1,14	1,14	1,63	1,56	1,05	1,35	hsa-miR-197
hsa-miR-199a-3p//hsa-miR-199b-3p	1,02	-2,38	2,53	1,19	2,12	-1,15	hsa-miR-199a-3p//hsa-miR-199b-3p
hsa-miR-199a-5p	-3,82	-6,20	-2,15	-2,37	1,10	-1,47	hsa-miR-199a-5p
hsa-miR-199b-5p	3,55	DOWN (3593)	6,00	-1,17	7,05	DOWN (1809)	hsa-miR-199b-5p
hsa-miR-19a	-1,96	DOWN (1088)	-3,34	-1,25	-2,69	DOWN (1494)	hsa-miR-19a
hsa-miR-19b	-1,19	-2,89	-3,45	-1,72	-2,00	-4,88	hsa-miR-19b
hsa-miR-200a	DOWN (3147)		DOWN (3147)		DOWN (3147)		hsa-miR-200a
hsa-miR-200b	DOWN (803)		DOWN (803)		DOWN (803)		hsa-miR-200b
hsa-miR-204	UP (1682)	UP (1488)				-1,13	hsa-miR-204
hsa-miR-20a	DOWN (9021)	1,29	-11,69	-1,12	-10,44	UP (1112)	hsa-miR-20a
hsa-miR-21	-1,81	-3,38	3,11	9,09	-2,93	-5,45	hsa-miR-21
hsa-miR-210	2,18	UP (986)	DOWN (2311)		DOWN (2311)	-5,10	hsa-miR-210
hsa-miR-214	1,19	-2,48	9,08	2,05	4,43	1,51	hsa-miR-214
hsa-miR-218	8,55	7,59	1,24	-1,69	2,09	1,86	hsa-miR-218
hsa-miR-22	-3,51	-1,08	-9,19	-1,08	-8,54	-2,62	hsa-miR-22
hsa-miR-22_star	DOWN (1013)		DOWN (1013)		DOWN (1013)		hsa-miR-22_star
hsa-miR-221	-2,69	-3,36	-4,04	-1,38	-2,92	-3,65	hsa-miR-221
hsa-miR-222	DOWN (2589)	DOWN (701)	-2,37	1,56	-3,70		hsa-miR-222
hsa-miR-223	3,96	1,05	2,73	2,90	-1,06	-3,98	hsa-miR-223
hsa-miR-23a	1,40	-1,22	1,66	2,42	-1,46	-2,49	hsa-miR-23a
hsa-miR-23b	-1,15	2,23	1,28	1,61	-1,26	2,04	hsa-miR-23b
hsa-miR-24	2,00	-1,43	2,65	-1,30	3,44	1,21	hsa-miR-24
hsa-miR-25	-3,26	-1,43	-1,45	-1,02	-1,43	1,60	hsa-miR-25
hsa-miR-26a	1,54	2,12	2,19	1,62	1,35	1,86	hsa-miR-26a
hsa-miR-26b	-1,27	1,83	3,48	-1,10	3,81	8,86	hsa-miR-26b
hsa-miR-27a	2,31	-1,67	2,85	1,82	1,56	-2,47	hsa-miR-27a
hsa-miR-27b	-1,35	2,32	1,25	-1,06	1,33	4,17	hsa-miR-27b
hsa-miR-28-3p		1,71	UP (816)	1,28	UP (639)	UP (1094)	hsa-miR-28-3p
hsa-miR-29a	1,03	1,22	2,77	1,10	2,52	3,00	hsa-miR-29a
hsa-miR-29b	2,11	1,44	1,28	-1,14	1,45	-1,01	hsa-miR-29b
hsa-miR-29c	-4,01	1,20	-6,61	-2,10	-3,14	1,53	hsa-miR-29c
hsa-miR-29c_star	DOWN (1160)		DOWN (1160)		DOWN (1160)		hsa-miR-29c_star
hsa-miR-301a	DOWN (914)		DOWN (914)		DOWN (914)		hsa-miR-301a
hsa-miR-30a	UP (1697)	-1,25	UP (2804)	-2,32	UP (6514)	3,07	hsa-miR-30a
hsa-miR-30a_star	DOWN (572)		DOWN (572)		DOWN (572)		hsa-miR-30a_star
hsa-miR-30b	2,37	-1,05	-2,31	-1,45	-1,59	-3,98	hsa-miR-30b
hsa-miR-30c	2,40	1,75	-1,42	-1,03	-1,37	-1,89	hsa-miR-30c
hsa-miR-30d	-1,50	-1,41	2,63	-1,31	3,45	3,68	hsa-miR-30d
hsa-miR-30e	-1,98	-1,44	-3,39	-1,78	-1,90	-1,38	hsa-miR-30e
hsa-miR-30e_star		DOWN (516)		DOWN (516)	UP (516)		hsa-miR-30e_star
hsa-miR-31	DOWN (5302)		DOWN (5302)		DOWN (5302)		hsa-miR-31
hsa-miR-320a	-4,27	-2,07	-4,14	-2,04	-2,03	1,02	hsa-miR-320a
hsa-miR-326		UP (528)	UP (555)	UP (555)		UP (528)	hsa-miR-326
hsa-miR-328		2,67	UP (617)	1,05	UP (589)	UP (1574)	hsa-miR-328
hsa-miR-331-3p	2,46	3,06	4,06	1,62	2,51	3,13	hsa-miR-331-3p
hsa-miR-335	UP (1144)	1,97		DOWN (519)	UP (519)	-1,12	hsa-miR-335
hsa-miR-337-3p	UP (592)					DOWN (592)	hsa-miR-337-3p
hsa-miR-338-3p	DOWN (857)		DOWN (857)		DOWN (857)		hsa-miR-338-3p
hsa-miR-339-5p	-1,17		DOWN (1621)		DOWN (1621)	DOWN (1380)	hsa-miR-339-5p
hsa-miR-33a	DOWN (695)		DOWN (695)		DOWN (695)		hsa-miR-33a
hsa-miR-342-3p	2,90	7,52	9,30	4,95	1,88	4,88	hsa-miR-342-3p
hsa-miR-345	UP (1103)	UP (933)				-1,18	hsa-miR-345
hsa-miR-34a	1,71	-1,78	-4,67	-1,68	-2,78	-8,51	hsa-miR-34a
hsa-miR-361-3p		UP (848)	UP (753)	UP (753)		UP (848)	hsa-miR-361-3p
hsa-miR-361-5p		UP (982)	UP (960)	UP (960)		UP (982)	hsa-miR-361-5p
hsa-miR-365		DOWN (621)	UP (979)	1,58	UP (621)		hsa-miR-365

Menigioma project

SOLiD
Next Generation Sequencing

microRNA profile

Name	P2-N vs P2-T	P1-N vs P1-T	P2-N vs P16-NN	P1-N vs P16-NN	P2-N vs P1-N	P2-T vs P1-T	Name
hsa-miR-374a			UP (565)	UP (565)			hsa-miR-374a
hsa-miR-374b		3,89	UP (1146)	1,55	UP (739)	UP (2873)	hsa-miR-374b
hsa-miR-376a	UP (4058)		UP (692)	UP (692)		DOWN (4058)	hsa-miR-376a
hsa-miR-376c	8,14	-1,93	4,16	2,10	1,98	-7,92	hsa-miR-376c
hsa-miR-378	1,08	UP (770)	-1,19	UP (591)	DOWN (704)	1,01	hsa-miR-378
hsa-miR-382	UP (642)					DOWN (642)	hsa-miR-382
hsa-miR-409-3p	1,17		1,27	UP (727)	DOWN (572)	DOWN (673)	hsa-miR-409-3p
hsa-miR-421		UP (692)				UP (692)	hsa-miR-421
hsa-miR-423-3p		1,41	UP (1069)	2,10	UP (508)	UP (718)	hsa-miR-423-3p
hsa-miR-423-5p			UP (668)	UP (668)			hsa-miR-423-5p
hsa-miR-424	6,33	2,01	1,38	1,66	-1,20	-3,79	hsa-miR-424
hsa-miR-425	1,04	-1,24	1,04	1,44	-1,39	-1,80	hsa-miR-425
hsa-miR-4286	1,83	1,73	-2,49	2,19	-5,44	-5,74	hsa-miR-4286
hsa-miR-4454	DOWN (8074)	UP (907)	-4,54	UP (1778)	DOWN (8074)	UP (907)	hsa-miR-4454
hsa-miR-4488	DOWN (1175)		DOWN (1175)		DOWN (1175)		hsa-miR-4488
hsa-miR-4497	DOWN (1582)		DOWN (1582)		DOWN (1582)		hsa-miR-4497
hsa-miR-451	1,90	-10,07	1,62	-1,71	2,78	-6,90	hsa-miR-451
hsa-miR-4516	DOWN (5047)		DOWN (5047)		DOWN (5047)		hsa-miR-4516
hsa-miR-4532	DOWN (2844)		DOWN (2844)		DOWN (2844)		hsa-miR-4532
hsa-miR-455-3p			UP (839)	UP (839)			hsa-miR-455-3p
hsa-miR-483-3p	UP (511)					DOWN (511)	hsa-miR-483-3p
hsa-miR-484	1,51	1,73	2,45	1,97	1,25	1,43	hsa-miR-484
hsa-miR-486-5p	DOWN (2281)	DOWN (2130)	-1,64	-1,53	-1,07		hsa-miR-486-5p
hsa-miR-487b	UP (714)					DOWN (714)	hsa-miR-487b
hsa-miR-495		UP (818)	UP (627)	UP (627)		UP (818)	hsa-miR-495
hsa-miR-497	UP (1124)	DOWN (1216)		DOWN (1216)	UP (1216)	DOWN (1124)	hsa-miR-497
hsa-miR-532-5p	UP (799)	1,47	UP (3531)	1,90	UP (1859)	3,42	hsa-miR-532-5p
hsa-miR-574-3p	-3,88	2,74	3,46	2,81	1,23	13,12	hsa-miR-574-3p
hsa-miR-574-5p	-4,86	DOWN (1762)	-2,64	-1,61	-1,64	DOWN (596)	hsa-miR-574-5p
hsa-miR-590-5p	UP (504)					DOWN (504)	hsa-miR-590-5p
hsa-miR-654-3p			UP (596)	UP (596)			hsa-miR-654-3p
hsa-miR-660	UP (712)					DOWN (712)	hsa-miR-660
hsa-miR-708	UP (642)		UP (512)	UP (512)		DOWN (642)	hsa-miR-708
hsa-miR-720	DOWN (914)		DOWN (914)		DOWN (914)		hsa-miR-720
hsa-miR-744		UP (551)				UP (551)	hsa-miR-744
hsa-miR-874		UP (822)	UP (839)	UP (839)		UP (822)	hsa-miR-874
hsa-miR-92a	-1,63	-1,45	-1,10	1,78	-1,97	-1,75	hsa-miR-92a
hsa-miR-93	UP (1448)	DOWN (1339)		DOWN (1339)	UP (1339)	DOWN (1448)	hsa-miR-93
hsa-miR-98	-1,37	UP (915)	DOWN (1489)		DOWN (1489)	-1,19	hsa-miR-98
hsa-miR-99a	5,43	-1,31	-1,78	-2,07	1,17	-6,09	hsa-miR-99a
hsa-miR-99b		2,17	UP (2070)	1,26	UP (1642)	UP (3558)	hsa-miR-99b

Discarded miRNAs with raw-read less than 50
Cutoff after normalization is 500 reads/million

Red = Overexpression in T vs N
Blue = Suppersion in T vs N

Down = Detected in N, not in T
UP = Detected in T, not in N
Detected values (pr. Million) in brackets

Table S2b. Summary of normalized (by totals) SOLiD miRNA reads, values in reads pr. million. For total miRNAs from meningioma tumors (T) or dura (N or NN). P, patient (numbers as in Table 1).

miRNA	P no. 16 NN	P no. 23* NN	P no. 2 N	P no. 2 T	P no. 1 N	P no. 1 T
hsa-let-7a	14763	14316	18985	19597	16719	21396
hsa-let-7a_star	208		351	130	154	224
hsa-let-7a-2_star	18				6	26
hsa-let-7b	13218	23120	18871	6458	14606	6277
hsa-let-7b_star	219		1289	74	127	243
hsa-let-7c	1147		2424	2236	1828	718
hsa-let-7d	3445		1385	1953	1908	4170
hsa-let-7d_star	410				161	138
hsa-let-7e	1346		662	3223	1311	1854
hsa-let-7e_star	51				18	54
hsa-let-7f	11591	5183	8328	21310	11867	22788
hsa-let-7f-1_star	57		186		20	47
hsa-let-7f-2_star	27		228		33	42
hsa-let-7g	11438		3773	39528	11849	16564
hsa-let-7g_star	31				17	22
hsa-let-7i	187		1052	910	1167	169
hsa-let-7i_star	461			204	285	136
hsa-miR-1	58				350	33
hsa-miR-100	14501	19171	18683	31635	26691	43785
hsa-miR-100_star						16
hsa-miR-101	2315		1118	7305	4178	2980
hsa-miR-101_star	6		174	42	79	48
hsa-miR-103a	66298	9051	16360	65055	35222	90773
hsa-miR-103a-2_star	15			73	21	45
hsa-miR-106a	80		171		63	18
hsa-miR-106b	768		294	346	613	525
hsa-miR-106b_star	334		189	74	196	114
hsa-miR-107	3037		1711	7813	2336	4455
hsa-miR-10a	210		1205		157	57
hsa-miR-10b	1601		1088	332	1576	45
hsa-miR-10b_star					9	
hsa-miR-1179					9	8
hsa-miR-1180	50				33	66
hsa-miR-1185	15			70	9	7
hsa-miR-122	24	66562	283989	3482	115	
hsa-miR-122_star			2401	41		
hsa-miR-1226	23			64	14	25
hsa-miR-1227	16				11	
hsa-miR-1228	9					11
hsa-miR-124	25				33	9
hsa-miR-1246	13		177			13
hsa-miR-1247	50				249	26
hsa-miR-1248	23				31	14
hsa-miR-1249	46			61	13	27
hsa-miR-125a-3p	21				13	20
hsa-miR-125a-5p	28497	11272	13243	30318	30475	71639
hsa-miR-125b	82386	67797	44735	104527	78409	180627
hsa-miR-125b-1_star	32				58	55
hsa-miR-125b-2_star	62			41	53	38
hsa-miR-126	24087		7016	3775	39252	10386
hsa-miR-126_star	19989	4196	6066	3775	22332	4317
hsa-miR-1260	1295		1774	1873	280	698
hsa-miR-1260b	1566		2601	1956	371	830

miRNA	P no. 16 NN	P no. 23* NN	P no. 2 N	P no. 2 T	P no. 1 N	P no. 1 T
hsa-miR-127-3p	180			279	46	56
hsa-miR-127-5p	49			129	17	24
hsa-miR-1270					19	10
hsa-miR-1271	52				48	159
hsa-miR-1275	8		225		28	40
hsa-miR-128	565		2520	341	276	664
hsa-miR-1280	235		494	91	47	165
hsa-miR-1285	7				8	
hsa-miR-1287	29			73	12	61
hsa-miR-129-3p	15				8	82
hsa-miR-1290	8				6	
hsa-miR-1291					21	7
hsa-miR-1296	11				17	7
hsa-miR-1299	6				6	9
hsa-miR-1301	39				30	73
hsa-miR-130a	6341		2107	13311	13666	13901
hsa-miR-130b	576		177	111	456	237
hsa-miR-130b_star	107		201		28	65
hsa-miR-132	121			99	80	163
hsa-miR-132_star	61			95	52	148
hsa-miR-133a					20	
hsa-miR-134	581		201	225	266	307
hsa-miR-1343	20				5	29
hsa-miR-135a			306		21	
hsa-miR-135b	8				11	7
hsa-miR-136	45			1500	179	164
hsa-miR-136_star	58			111	52	39
hsa-miR-138	76		839		120	1400
hsa-miR-138-1_star	8		189		14	155
hsa-miR-139-3p					7	
hsa-miR-139-5p	1626		893	179	367	182
hsa-miR-140-3p	10653	10778	4001	3940	25987	9205
hsa-miR-140-5p	1437		902	1401	4596	1098
hsa-miR-141	10		183		13	10
hsa-miR-142-3p	998		692	905	1118	149
hsa-miR-142-5p	351		974	141	392	52
hsa-miR-143	6725	4278	5424	3116	10714	2071
hsa-miR-143_star	18					
hsa-miR-144	175		1310	1673	3042	75
hsa-miR-144_star	350		2733	152	796	120
hsa-miR-145	47176	16126	23271	9475	31451	14892
hsa-miR-145_star	90		255	45	107	17
hsa-miR-146a	383		285	998	621	95
hsa-miR-146b-3p	10				22	17
hsa-miR-146b-5p	415		291	207	484	468
hsa-miR-147b	8				20	7
hsa-miR-148a	2829		2820	7097	2367	6551
hsa-miR-148a_star	7					13
hsa-miR-148b	402	6253	6380	1061	287	255
hsa-miR-148b_star	6				8	7
hsa-miR-149	201		587	42	144	441
hsa-miR-150	4784		689	3160	1445	580
hsa-miR-151-3p	620		183	157	1271	1034
hsa-miR-151-5p	3570		668	2074	4051	5796
hsa-miR-151b					7	10
hsa-miR-152	1622		899	5672	1878	2130
hsa-miR-153	21				67	33
hsa-miR-154	93			99	47	42

miRNA	P no. 16 NN	P no. 23* NN	P no. 2 N	P no. 2 T	P no. 1 N	P no. 1 T
hsa-miR-154_star	74			93	9	22
hsa-miR-155	35			64	7	7
hsa-miR-15a	1917		2296	553	1189	1100
hsa-miR-15a_star					17	29
hsa-miR-15b	6385	5101	2886	1216	2023	1555
hsa-miR-15b_star	80		818		319	62
hsa-miR-16	3597		1807	125	2026	1430
hsa-miR-16-2_star	398		1627	76	865	237
hsa-miR-17	2140	10285	21251	2054	3894	2796
hsa-miR-17_star	64		381	337	103	114
hsa-miR-181a	25422		2691	8285	4306	14250
hsa-miR-181a_star	191		210	127	121	416
hsa-miR-181a-2_star	35			99	164	99
hsa-miR-181b	1559		674	881	634	1012
hsa-miR-181c	277			386	186	792
hsa-miR-181c_star	8			45	10	61
hsa-miR-181d	26				7	32
hsa-miR-182	307		468		87	38
hsa-miR-183	56				29	9
hsa-miR-185	3394	11601	4957	1954	2946	1564
hsa-miR-185_star					5	
hsa-miR-186	1538		551	615	2535	1288
hsa-miR-186_star	11				18	11
hsa-miR-187	79				12	
hsa-miR-188-3p	8				6	
hsa-miR-188-5p	90			61	65	42
hsa-miR-18a	156		438		51	56
hsa-miR-18a_star	18				12	8
hsa-miR-18b	19				11	
hsa-miR-190	68			133	182	267
hsa-miR-190b					8	
hsa-miR-191	15255	6089	6731	9679	12564	33379
hsa-miR-191_star						11
hsa-miR-192	1086		539	456	2346	4020
hsa-miR-192_star					9	9
hsa-miR-193a-3p	164		435	491	252	66
hsa-miR-193a-5p	864				130	164
hsa-miR-193b	3272		1304	98	2971	514
hsa-miR-193b_star	83				333	47
hsa-miR-194	78		183	58	140	102
hsa-miR-195	4378		435	781	5993	1879
hsa-miR-195_star	38				89	27
hsa-miR-196a	7				5	7
hsa-miR-196b					8	
hsa-miR-197	1015		623	549	651	741
hsa-miR-1973	9				7	
hsa-miR-199a-3p//hsa-miR-199b-3p	40563	27398	16045	16436	34066	14294
hsa-miR-199a-5p	15187	117657	32612	8547	36026	5811
hsa-miR-199b-5p	3059		509	1809	3593	312
hsa-miR-19a	874		2922	1494	1088	434
hsa-miR-19a_star					5	
hsa-miR-19b	3306	10367	11418	9620	5700	1972
hsa-miR-19b-1_star	9				17	19
hsa-miR-200a		5101	3147	114		
hsa-miR-200b			803			
hsa-miR-200c	19		159		10	16
hsa-miR-202_star					6	

miRNA	P no. 16 NN	P no. 23* NN	P no. 2 N	P no. 2 T	P no. 1 N	P no. 1 T
hsa-miR-203	26	4608	183		105	26
hsa-miR-204	66	9380		1682	377	1488
hsa-miR-205	12	19171		83	6	
hsa-miR-206					19	
hsa-miR-20a	772		9021	336	864	1112
hsa-miR-20a_star	20				28	61
hsa-miR-20b	349			83	331	105
hsa-miR-21	35992		11583	6383	3958	1171
hsa-miR-21_star	113				24	
hsa-miR-210	108		2311	5030	240	986
hsa-miR-2110	14				24	17
hsa-miR-2114					6	7
hsa-miR-2114_star						8
hsa-miR-2116_star	13					
hsa-miR-212	12				8	14
hsa-miR-214	24155	12753	2661	3158	11793	4759
hsa-miR-214_star	33				48	21
hsa-miR-215	15		258		18	10
hsa-miR-218	1730		1394	11912	2917	22136
hsa-miR-22	1826	12753	16786	4784	1966	1826
hsa-miR-22_star	354		1013	94	59	55
hsa-miR-221	2172		8766	3264	3006	895
hsa-miR-221_star	28				17	
hsa-miR-222	1092		2589	231	701	66
hsa-miR-223	11400	7487	4169	16501	3933	4141
hsa-miR-223_star	14				23	
hsa-miR-224	88			45	27	18
hsa-miR-224_star	84			240	52	97
hsa-miR-2355-3p	11				12	
hsa-miR-2355-5p					16	8
hsa-miR-23a	20610	10038	12440	17415	8517	6985
hsa-miR-23a_star	13					
hsa-miR-23b	8880		6932	6010	5504	12250
hsa-miR-23b_star	10				7	13
hsa-miR-24	36173	7487	13630	27275	46928	32927
hsa-miR-24-1_star	12				55	35
hsa-miR-24-2_star	263			121	230	87
hsa-miR-25	1612		2344	718	1644	1149
hsa-miR-26a	37546	8310	17121	26328	23183	49049
hsa-miR-26a-1_star	16				10	12
hsa-miR-26a-2_star	21				43	21
hsa-miR-26b	6165		1771	1393	6754	12337
hsa-miR-26b_star	54			85	50	145
hsa-miR-27a	5610		1969	4556	3078	1842
hsa-miR-27a_star	30				33	
hsa-miR-27b	1786		1424	1057	1895	4405
hsa-miR-27b_star	87			42	92	130
hsa-miR-28-3p	816		225		639	1094
hsa-miR-28-5p	381			116	173	339
hsa-miR-296-3p					12	19
hsa-miR-296-5p	113				43	301
hsa-miR-299-3p	62				5	32
hsa-miR-299-5p	216			78	42	83
hsa-miR-29a	12908		4663	4782	11751	14356
hsa-miR-29a_star	37			40	129	56
hsa-miR-29b	16086		12608	26577	18305	26358
hsa-miR-29b-1_star	39				10	43
hsa-miR-29b-2_star	59			81	27	94

miRNA	P no. 16 NN	P no. 23* NN	P no. 2 N	P no. 2 T	P no. 1 N	P no. 1 T
hsa-miR-29c	4252	14810	28108	7011	8942	10750
hsa-miR-29c_star	147		1160	295	326	382
hsa-miR-301a	146		914	443	122	307
hsa-miR-301b	14				11	7
hsa-miR-3065-3p					8	7
hsa-miR-3065-5p					6	10
hsa-miR-30a	2804		438	1697	6514	5206
hsa-miR-30a_star	179		572	182	308	413
hsa-miR-30b	3285	7734	7594	17976	4763	4519
hsa-miR-30c	5930	7405	8400	20163	6128	10694
hsa-miR-30c-1_star					7	8
hsa-miR-30c-2_star					18	35
hsa-miR-30d	2839		1079	718	3718	2644
hsa-miR-30d_star	6				14	10
hsa-miR-30e	1010		3422	1725	1801	1253
hsa-miR-30e_star	364		183	300	516	423
hsa-miR-31	402		5302	43	459	215
hsa-miR-31_star					5	
hsa-miR-3152-5p					6	
hsa-miR-3154					16	11
hsa-miR-3182	22				24	11
hsa-miR-3194-5p					5	
hsa-miR-3196	20		192			
hsa-miR-32	72			78	103	112
hsa-miR-32_star	26				28	52
hsa-miR-3200-3p					6	18
hsa-miR-320a	2325	8557	9614	2254	4737	2292
hsa-miR-320b	78			40	154	40
hsa-miR-320c	22				72	23
hsa-miR-320d	9				48	11
hsa-miR-323-3p	105			85	18	135
hsa-miR-323b-3p	87					42
hsa-miR-324-3p					7	
hsa-miR-324-5p	186			429	102	140
hsa-miR-326	555		246	330	314	528
hsa-miR-328	617		324	291	589	1574
hsa-miR-329	147			164	35	132
hsa-miR-330-3p	144				13	72
hsa-miR-330-5p	6					
hsa-miR-331-3p	2483		611	1503	1535	4699
hsa-miR-331-5p	7					11
hsa-miR-335	313		168	1144	519	1022
hsa-miR-335_star	46					182
hsa-miR-337-3p	371		213	592	154	255
hsa-miR-337-5p	205			57	129	62
hsa-miR-338-3p	86		857	113	156	327
hsa-miR-338-5p	18				34	163
hsa-miR-339-3p	105				110	99
hsa-miR-339-5p	477		1621	1380	377	365
hsa-miR-33a	8		695	177	53	140
hsa-miR-33a_star	21			54	30	161
hsa-miR-33b	11			134	11	25
hsa-miR-33b_star	26				18	41
hsa-miR-340	147			48	63	121
hsa-miR-340_star	103			65	38	266
hsa-miR-342-3p	5186		557	1617	1049	7887
hsa-miR-342-5p	9				18	10
hsa-miR-345	350		396	1103	369	933

miRNA	P no. 16 NN	P no. 23* NN	P no. 2 N	P no. 2 T	P no. 1 N	P no. 1 T
hsa-miR-34a	3208	5348	14993	25689	5387	3018
hsa-miR-34a_star	55			195	55	49
hsa-miR-34b_star	7			45	7	18
hsa-miR-34c-5p	7			337	29	31
hsa-miR-3605-3p	14				7	14
hsa-miR-3605-5p	6				5	11
hsa-miR-3607-5p	13					7
hsa-miR-3609					5	
hsa-miR-361-3p	753			62	404	848
hsa-miR-361-5p	960			425	430	982
hsa-miR-3613-5p	40				34	23
hsa-miR-3615	35					
hsa-miR-3616-3p						15
hsa-miR-3617						17
hsa-miR-362-3p	86			98	93	36
hsa-miR-362-5p	186				52	66
hsa-miR-3620	14					22
hsa-miR-3622a-5p	8				11	20
hsa-miR-363	109			68	94	37
hsa-miR-3647-3p					14	
hsa-miR-365	979		249	122	621	376
hsa-miR-3651					5	
hsa-miR-3656	18		294		5	23
hsa-miR-3679-5p	8				5	9
hsa-miR-369-3p	226			89	53	56
hsa-miR-369-5p	76			69	40	55
hsa-miR-370	394			201	91	167
hsa-miR-374a	565			74	453	267
hsa-miR-374a_star	13				22	22
hsa-miR-374b	1146		333	97	739	2873
hsa-miR-374b_star	8				5	33
hsa-miR-374c	6					20
hsa-miR-376a	692		300	4058	478	136
hsa-miR-376a_star	86			176	44	33
hsa-miR-376b	57			254	19	
hsa-miR-376c	2369		569	4634	1129	585
hsa-miR-377	20				13	12
hsa-miR-377_star	12				6	10
hsa-miR-378	591		704	758	319	770
hsa-miR-378_star	82			91	48	83
hsa-miR-378c	23			52	16	31
hsa-miR-378d					12	7
hsa-miR-378f						8
hsa-miR-379	324			334	129	300
hsa-miR-379_star	22					16
hsa-miR-380	25					12
hsa-miR-381	138			112	40	62
hsa-miR-382	375		168	642	95	151
hsa-miR-383					5	
hsa-miR-3909	9					
hsa-miR-3928					10	
hsa-miR-3940-3p	15					12
hsa-miR-409-3p	727		572	673	237	399
hsa-miR-409-5p	66			85		20
hsa-miR-410	64				9	12
hsa-miR-411	8					8
hsa-miR-411_star	37				7	20
hsa-miR-421	396		369	86	118	692

miRNA	P no. 16 NN	P no. 23* NN	P no. 2 N	P no. 2 T	P no. 1 N	P no. 1 T
hsa-miR-423-3p	1069		306	444	508	718
hsa-miR-423-5p	668		174	268	483	291
hsa-miR-424	1860		1346	8523	1119	2249
hsa-miR-424_star	10					16
hsa-miR-425	1289		1244	1297	894	720
hsa-miR-425_star	53			79	72	102
hsa-miR-4284	7					17
hsa-miR-4286	1167	4361	2901	5295	533	922
hsa-miR-4306					5	
hsa-miR-431	48			54		
hsa-miR-432	88			142	13	15
hsa-miR-432_star	6					
hsa-miR-433	33			68		
hsa-miR-4423-3p					7	44
hsa-miR-4423-5p						70
hsa-miR-4454	1778	24190	8074	359	258	907
hsa-miR-4488			1175			12
hsa-miR-4497			1582			
hsa-miR-449a					8	
hsa-miR-450a	39				11	74
hsa-miR-450b-5p	12				7	28
hsa-miR-451	113826	352065	70241	133577	194980	19357
hsa-miR-4516	50		5047		26	18
hsa-miR-452	67			393	85	31
hsa-miR-4532	35		2844		45	23
hsa-miR-454	25					25
hsa-miR-454_star	15				14	42
hsa-miR-455-3p	839			79	311	92
hsa-miR-455-5p	18				48	
hsa-miR-4632	8					
hsa-miR-4683	7				14	
hsa-miR-4717-5p					4	
hsa-miR-4728-3p	15				21	22
hsa-miR-4731-3p	6					
hsa-miR-4732-3p	8					
hsa-miR-4732-5p					9	
hsa-miR-4772-3p	9					
hsa-miR-4787-3p	7				7	
hsa-miR-4792					14	
hsa-miR-4800-5p					5	
hsa-miR-483-3p	52			511	18	143
hsa-miR-483-5p	28			372	66	40
hsa-miR-484	4300		1753	2646	2188	3777
hsa-miR-485-3p	154			51	34	41
hsa-miR-485-5p	38				11	20
hsa-miR-486-3p	70				77	
hsa-miR-486-5p	1390		2281	48	2130	423
hsa-miR-487a	68			149	9	29
hsa-miR-487b	377		150	714	78	310
hsa-miR-489	60			137	39	38
hsa-miR-491-5p	10				46	18
hsa-miR-493	26			57	7	23
hsa-miR-493_star	95				7	22
hsa-miR-494	424			216	67	275
hsa-miR-495	627			281	105	818
hsa-miR-496	17					
hsa-miR-497	482		321	1124	1216	184
hsa-miR-497_star					19	

miRNA	P no. 16 NN	P no. 23* NN	P no. 2 N	P no. 2 T	P no. 1 N	P no. 1 T
hsa-miR-499-5p					11	15
hsa-miR-500a	64				16	11
hsa-miR-500a_star	135				33	34
hsa-miR-501-3p	14				6	
hsa-miR-501-5p	42				15	8
hsa-miR-502-3p	377			99	165	110
hsa-miR-503	24			50	13	50
hsa-miR-504	19					
hsa-miR-505	350		159	106	75	168
hsa-miR-505_star	41				35	61
hsa-miR-511	32				6	
hsa-miR-517a					6	
hsa-miR-532-3p	298				43	104
hsa-miR-532-5p	3531		444	799	1859	2731
hsa-miR-539	71				24	32
hsa-miR-542-3p	81			229	75	280
hsa-miR-542-5p	32			59	16	118
hsa-miR-543	325			62	12	144
hsa-miR-545	7			58	7	
hsa-miR-545_star					5	
hsa-miR-548aa	55		183		40	92
hsa-miR-548d-3p	6					
hsa-miR-548v						15
hsa-miR-549	8				7	15
hsa-miR-550a	37				33	26
hsa-miR-550a_star	62				39	18
hsa-miR-551a	9					
hsa-miR-551b	37				57	9
hsa-miR-570					15	
hsa-miR-574-3p	23109		6671	1719	8232	22557
hsa-miR-574-5p	1098		2898	596	1762	266
hsa-miR-576-5p	15				22	9
hsa-miR-579					5	
hsa-miR-582-5p	14				11	
hsa-miR-584	16				5	
hsa-miR-589	9				7	
hsa-miR-589_star	12				20	15
hsa-miR-590-3p	157		189		185	254
hsa-miR-590-5p	125		210	504	396	227
hsa-miR-598	41			131	7	76
hsa-miR-616					5	
hsa-miR-616_star	11				5	16
hsa-miR-618	7					
hsa-miR-624_star	16				7	
hsa-miR-625	9				12	73
hsa-miR-625_star	94				43	165
hsa-miR-627	25				70	15
hsa-miR-628-3p	12				7	31
hsa-miR-628-5p	52				100	378
hsa-miR-629	33				18	28
hsa-miR-629_star						8
hsa-miR-636					6	
hsa-miR-641	9				16	31
hsa-miR-642a						15
hsa-miR-642b						16
hsa-miR-652	13					14
hsa-miR-654-3p	596		201	409	111	488
hsa-miR-655	71			40	47	72

miRNA	P no. 16 NN	P no. 23* NN	P no. 2 N	P no. 2 T	P no. 1 N	P no. 1 T
hsa-miR-656	44				9	27
hsa-miR-660	331			712	266	161
hsa-miR-664	238			53	113	395
hsa-miR-664_star	26				41	24
hsa-miR-671-3p	52				5	72
hsa-miR-671-5p	40			157	215	53
hsa-miR-675	13				5	
hsa-miR-7	102		273	72	57	43
hsa-miR-7-1_star	78			40	99	85
hsa-miR-7-2_star	10				5	12
hsa-miR-708	512		222	642	449	317
hsa-miR-708_star	11				34	9
hsa-miR-720	442		914	142	51	173
hsa-miR-744	413			163	148	551
hsa-miR-744_star	11				11	22
hsa-miR-766	99			46	23	297
hsa-miR-769-3p	30				19	13
hsa-miR-769-5p	38			44	56	63
hsa-miR-873				54	32	181
hsa-miR-874	839		198	86	371	822
hsa-miR-876-3p					10	27
hsa-miR-876-5p					16	166
hsa-miR-877					19	
hsa-miR-877_star	10				11	33
hsa-miR-887	230			268	376	451
hsa-miR-889	11					
hsa-miR-9					8	
hsa-miR-92a	4400		4846	2969	2466	1701
hsa-miR-92a-1_star					6	7
hsa-miR-92b	297			45	141	246
hsa-miR-92b_star	12				14	15
hsa-miR-93	500		336	1448	1339	185
hsa-miR-93_star	101			102	41	86
hsa-miR-935	27				17	65
hsa-miR-940	58				7	23
hsa-miR-941	32				20	14
hsa-miR-95	153				31	84
hsa-miR-96	49				21	
hsa-miR-98	244		1489	1091	467	915
hsa-miR-99a	7707	31841	13690	74329	15968	12215
hsa-miR-99a_star	270				254	335
hsa-miR-99b	2070		423	81	1642	3558
hsa-miR-99b_star	222			108	57	255

* This patient sample NN Dura was used for sequencing, but not for validation by RT-qPCR

Table S2c. Global comparisons of all expressed miRNAs between grades I and II meningioma as well as between dura controls N (patient with a tumor grade I or II) and NN (patient without tumors).

**Sample**	**T-TEST**
Grade I vs. Grade II	0,9809
Normal (N) (from patient with grade I) vs Normal (N) (from patient with grade II)	0,7027
Normal (NN) vs Normal (N) (from patient with grade II)	0,9315
Normal (N) (from patient with grade I) vs Normal (NN)	0,6953
Normal (N) (from patient with grade I) vs Tumor (Grade I)	0,9479
Normal N (from patient with grade II) vs Tumor (Grade II)	0,7192

Table S3a. miRNA RT-qPCR analysis. The ΔΔCq method was used to calculate all the fold changes and the Student's t-test (two tailed) was used to calculate all the p-values.

Patient	Grade	miR-574-3p	let-7g	miR-17	miR-26b	miR-218	miR-376c	miR-34a	miR-342-3p	miR-424	miR-143	miR-130a	miR-152	miR-199a-5p	miR-99a	miR-148b	miR-193b	miR-451	miR-21	miR-200a
2	II	-0,798	-4,728	2,153	-1,618	-0,968	0,500	-3,999	-1,198	-3,169	-2,278	-0,343	-2,261	-1,886	-3,653	-0,900	1,708	-4,225	-3,074	7,256
1	I	-0,497	-4,365	2,097	-2,875	-2,311	1,207	-3,202	-0,025	-3,066	-2,612	-0,715	-1,237	-1,681	-2,684	0,267	0,833	-4,358	-1,908	8,133
3	II	-0,376	-4,341	2,596	-2,192	-0,925	1,041	-3,786	-0,184	-2,627	-1,931	-0,975	-1,722	-3,354	-3,196	-0,494	1,448	-3,651	-2,041	8,636
4	I	-0,546	-4,912	2,402	-1,873	-1,731	-1,770	-4,046	-1,016	-4,412	-4,213	-0,644	-2,893	-0,881	-2,225	-1,093	-1,778	-4,241	-3,030	10,548
5	I	-1,248	-4,696	2,281	-2,074	-1,015	0,481	-3,789	-0,957	-1,933	-2,769	-0,746	-2,580	-3,468	-3,728	-0,660	0,063	-5,828	-2,905	8,502
6	II	-0,449	-4,366	2,485	-2,540	-0,828	2,229	-1,521	0,752	-1,336	-2,821	-0,551	-2,168	-2,245	-3,753	-0,715	-0,831	-4,742	-5,433	8,956
7	I	0,036	-4,010	3,360	-2,530	-3,007	0,831	-3,543	0,190	-4,514	-2,855	0,597	-1,727	-1,027	-2,411	-0,156	-1,506	-5,742	-3,299	7,560
8	I	-1,343	-4,429	2,564	-2,049	-1,376	0,297	-3,365	-0,920	-2,411	-2,821	-0,985	-2,126	-3,040	-4,174	0,009	0,967	-2,612	-2,733	7,690
9	I	-1,415	-4,476	2,640	-1,957	-0,923	0,921	-3,635	-0,619	-2,442	-2,472	-0,280	-2,547	-3,348	-4,369	-0,590	-0,104	-2,447	-3,008	8,088
10	I	0,670	-4,118	2,453	-2,119	-1,452	2,085	-2,394	0,654	-1,927	-2,396	-0,611	-1,825	1,100	-1,937	-0,261	-0,166	-4,494	-3,780	9,501
11	I	0,030	-3,988	1,977	-2,078	-0,783	2,731	-1,219	-0,277	-4,284	-3,246	0,451	-2,082	-2,068	-3,677	0,149	0,316	-6,460	-3,200	8,863
12	I	-1,429	-4,522	2,516	-1,816	-0,971	-0,173	-3,194	-0,919	-3,336	-2,823	-1,165	-2,659	-3,193	-5,062	-0,771	2,106	-3,292	-3,719	7,043
13	I	-1,123	-4,883	1,249	-2,582	-3,275	1,277	-3,254	-0,792	-2,762	-4,278	-0,091	-1,873	-3,226	-2,726	-0,286	-0,610	-5,643	-2,732	7,815
14	I	-0,625	-3,033	3,358	-1,051	-0,407	1,002	-2,827	-0,357	-1,122	-1,435	0,181	-1,380	-2,959	-3,773	1,027	-0,066	-0,643	-1,075	9,233
15	I	-1,000	-3,775	2,427	-2,096	2,782	1,948	-1,492	0,080	-1,911	-5,464	1,720	-1,230	-4,321	-4,676	0,724	-3,150	-8,902	-4,134	5,385
9	N	-1,162	-4,516	1,041	-2,043	1,025	2,113	-0,716	-0,135	-1,361	-4,297	1,184	-1,143	-2,993	-4,137	0,254	-2,717	-9,711	-4,156	8,309
10	N	-0,805	-4,221	1,076	-2,060	0,777	1,485	-2,005	-0,002	-1,755	-4,956	0,662	-0,783	-3,115	-2,508	0,216	-1,882	-9,131	-4,943	9,147
13	N	-1,074	-3,869	1,679	-2,142	1,227	1,610	-0,942	-0,157	-1,637	-5,328	0,663	-1,097	-3,175	-3,979	0,520	-2,441	-8,364	-4,246	7,660
15	N	-1,049	-4,007	1,343	-2,136	1,135	1,595	-0,979	-0,104	-2,185	-5,153	0,880	-0,769	-3,024	-4,002	0,449	-2,276	-8,864	-4,467	8,629
16	NN	-1,092	-4,043	1,734	-1,059	0,913	0,350	-2,208	-0,482	-2,548	-4,826	1,089	-0,565	-2,708	-1,969	0,340	-1,054	-7,385	-7,004	8,561

	miR-574-3p	let-7g	miR-17	miR-26b	miR-218	miR-376c	miR-34a	miR-342-3p	miR-424	miR-143	miR-130a	miR-152	miR-199a-5p	miR-99a	miR-148b	miR-193b	miR-451	miR-21	miR-200a
Average T	-0,67	-4,31	2,44	-2,10	-1,15	0,97	-3,02	-0,37	-2,75	-2,96	-0,28	-2,02	-2,37	-3,47	-0,25	-0,05	-4,49	-3,07	8,21
Average N	-1,04	-4,13	1,37	-1,89	1,02	1,43	-1,37	-0,18	-1,90	-4,91	0,90	-0,87	-3,00	-3,32	0,36	-2,07	-8,69	-4,96	8,46
Fold Change	-1,28562	1,13160	**-2,08868**	1,15565	**4,47300**	1,37223	**3,13323**	1,14613	1,80630	**-3,86719**	**2,25423**	2,21845	-1,54752	1,11004	1,52157	**-4,06329**	**-18,45249**	**-3,71039**	1,18696
P-value	0,21164	0,44390	**0,00040**	0,37653	**0,00280**	0,39450	**0,00208**	0,49589	0,10305	**0,00060**	**0,00356**	0,00016	0,32484	0,76006	0,04109	**0,00658**	**0,00021**	**0,00267**	0,66813
Std.av T	0,610	0,482	0,510	0,440	1,369	1,095	0,939	0,614	1,061	1,010	0,758	0,517	1,363	0,921	0,600	1,404	1,944	1,013	1,212
Std.av N	0,136	0,249	0,325	0,465	0,178	0,651	0,684	0,181	0,470	0,393	0,240	0,243	0,180	1,006	0,128	0,645	0,877	1,181	0,542
Max T	0,670	-3,033	3,360	-1,051	2,782	2,731	-1,219	0,752	-1,122	-1,435	1,720	-1,230	1,100	-1,937	1,027	2,106	-0,643	-1,075	10,548
Min T	-1,429	-4,912	1,249	-2,875	-3,275	-1,770	-4,046	-1,198	-4,514	-5,464	-1,165	-2,893	-4,321	-5,062	-1,093	-3,150	-8,902	-5,433	5,385
Max N	-0,805	-3,869	1,734	-1,059	1,227	2,113	-0,716	-0,002	-1,361	-4,297	1,184	-0,565	-2,708	-1,969	0,520	-1,054	-7,385	-4,156	9,147
Min N	-1,162	-4,516	1,041	-2,142	0,777	0,350	-2,208	-0,482	-2,548	-5,328	0,662	-1,143	-3,175	-4,137	0,216	-2,717	-9,711	-7,004	7,660

Table S3b. Relative expression of microRNAs of grade I versus grade II for the miRs that exhibited differential expression between tumors versus controls as shown in Table 3. Fold change between grades I and II and the p-values are shown. The ΔΔCq method was used to calculate all the fold changes and the Student's t-test (two tailed) was used to calculate all the p-values.

	**miR-21**	**miR-34a**	**miR-143**	**miR-193b**	**miR-218**
Average Grade I	-2,96	-3,00	-3,12	-0,26	-1,21
Average Grade II	-3,52	-3,10	-2,34	0,77	-0,91
Fold Change, I vs II	-1,47	-1,08	1,71	2,05	1,23
*P-value	0,768	0,839	0,986	0,809	0,471

*No significant difference between grade I and grade II in microRNA expression reported in Table 3.

Table S4a. Initial RT-qPCR mRNA data analysis of meningiomas compared to control. The ΔΔCq method was used to calculate all the fold changes and the Student's t-test (two tailed) was used to calculate all the p-values.

Patient	Grade	ACVR1C	CCNG1	E2F5	MTOR	PTEN_AB	RICTOR	RUNX1T1	SIRT1	TP53-FAM	TP63
2	II	NA	0,856	6,488	4,707	6,067	5,989	10,598	6,216	3,821	5,688
1	I	NA	1,286	6,369	5,825	6,961	7,000	9,597	5,677	4,289	4,300
3	II	NA	1,792	7,189	5,730	6,681	6,952	10,102	5,972	4,914	6,222
4	I	NA	2,263	5,411	6,225	7,083	6,029	7,488	5,725	4,078	4,013
5	I	NA	1,747	6,738	5,886	7,096	6,865	7,797	6,497	4,474	11,263
6	II	NA	4,260	6,266	7,249	9,289	7,706	NA	8,188	4,622	2,858
7	I	11,008	1,638	6,585	5,821	6,437	7,480	10,454	6,809	5,194	6,121
8	I	12,643	0,732	5,643	6,160	6,512	6,347	7,564	5,925	5,436	9,613
9	I	NA	2,685	6,641	5,983	6,279	6,698	9,402	5,823	5,160	8,523
10	I	NA	1,531	5,411	5,983	5,776	6,725	10,230	6,590	4,066	4,059
11	I	NA	1,195	5,535	6,237	5,913	6,577	NA	6,343	3,818	3,816
12	I	NA	1,918	6,565	6,818	6,632	6,245	10,648	6,289	4,632	4,863
13	I	NA	1,871	6,464	6,211	6,402	6,695	8,793	6,572	5,373	4,643
14	I	NA	3,971	7,023	6,845	6,946	7,375	NA	7,636	3,253	NA
15	I	NA	2,556	7,428	6,037	5,694	6,822	10,926	6,516	4,287	6,604
9	N	NA	NA	NA	NA	NA	NA	NA	NA	NA	NA
10	N	NA	1,016	NA	NA	NA	NA	NA	NA	2,436	NA
13	N	NA	2,140	5,576	5,295	5,036	5,090	6,661	4,678	3,809	NA
15	N	NA	0,841	4,565	NA	NA	5,098	NA	NA	3,020	NA
16	NN	NA	2,719	6,344	5,570	5,607	6,612	8,161	7,488	3,638	NA
	Average T	11,83	2,02	6,38	6,11	6,65	6,77	9,47	6,45	4,49	5,90
	Average N	#DIV/0!	1,68	5,49	5,43	5,32	5,60	7,41	6,08	3,23	#DIV/0!
	Fold Change	#DIV/0!	-1,26684	-1,85182	-1,60408	**-2,51336**	**-2,24544**	**-4,15693**	**-1,29144**	**-2,40909**	#DIV/0!
	P-value	#DIV/0!	0,55024	0,05237	0,12956	**0,05218**	**0,00465**	**0,05201**	**0,56949**	**0,00249**	#DIV/0!
	Std.av T	4,172	1,014	0,633	0,582	0,860	0,500	4,076	0,692	0,638	2,782
	Std.av N	0,000	1,083	3,075	2,977	2,922	3,129	4,094	3,477	1,541	0,000
	Max T	12,643	4,260	7,428	7,249	9,289	7,706	10,926	8,188	5,436	**11,263**
	Min T	11,008	0,732	5,411	4,707	5,694	5,989	7,488	5,677	3,253	**2,858**
	Max N	0,000	2,719	6,344	5,570	5,607	6,612	8,161	7,488	3,809	**0,000**
	Min N	0,000	0,841	4,565	5,295	5,036	5,090	6,661	4,678	2,436	**0,000**

Table S4b. Grade I vs Grade II in the Initial RT-qPCR mRNA data analysis of meningiomas grade I compared to grade II did not reveal a significant differences in the tested mRNAs using one reference gene. The ΔΔCq method was used to calculate all the fold changes and the Student's t-test (two tailed) was used to calculate all the p-values.

Patient	Grade	ACVR1C	CCNG1	E2F5	MTOR	PTEN_AB	RICTOR	RUNX1T1	SIRT1	TP53-FAM	TP63
1	I	NA	1,286	6,369	5,825	6,961	7,000	9,597	5,677	4,289	4,300
4	I	NA	2,263	5,411	6,225	7,083	6,029	7,488	5,725	4,078	4,013
5	I	NA	1,747	6,738	5,886	7,096	6,865	7,797	6,497	4,474	11,263
7	I	11,008	1,638	6,585	5,821	6,437	7,480	10,454	6,809	5,194	6,121
8	I	12,643	0,732	5,643	6,160	6,512	6,347	7,564	5,925	5,436	9,613
9	I	NA	2,685	6,641	5,983	6,279	6,698	9,402	5,823	5,160	8,523
11	I	NA	1,195	5,535	6,237	5,913	6,577	NA	6,343	3,818	3,816
12	I	NA	1,918	6,565	6,818	6,632	6,245	10,648	6,289	4,632	4,863
13	I	NA	1,871	6,464	6,211	6,402	6,695	8,793	6,572	5,373	4,643
14	I	NA	3,971	7,023	6,845	6,946	7,375	NA	7,636	3,253	NA
2	II	NA	0,856	6,488	4,707	6,067	5,989	10,598	6,216	3,821	5,688
3	II	NA	1,792	7,189	5,730	6,681	6,952	10,102	5,972	4,914	6,222
6	II	NA	4,260	6,266	7,249	9,289	7,706	NA	8,188	4,622	2,858
10	#REF!	NA	1,531	5,411	5,983	5,776	6,725	10,230	6,590	4,066	4,059
15	#REF!	NA	2,556	7,428	6,037	5,694	6,822	10,926	6,516	4,287	6,604
9	N	NA	NA	NA	NA	NA	NA	NA	NA	NA	NA
10	N	NN	1.016	NA	NA	NA	NA	NA	NA	2,436	NA
13	N	NA	2,140	5,576	5,295	5,036	5,090	6,661	4,678	3,809	NA
15	N	NA	0,841	4,565	NA	NA	5,098	NA	NA	3,020	NA
16	NN	NA	2,719	6,344	5,570	5,607	6,612	8,161	7,488	3,638	NA

	ACVR1C	CCNG1	E2F5	MTOR	PTEN_AB	RICTOR	RUNX1T1	SIRT1	TP53-FAM	TP63
Average Grade 1	11,83	1,93	6,30	6,20	6,63	6,73	8,97	6,33	4,57	6,35
Average Grade 2	#DIV/0!	2,30	6,65	5,90	7,35	6,88	10,35	6,79	4,45	4,92
Fold Change	#DIV/0!	1,29447	1,27469	-1,23575	1,64623	1,11056	2,60631	1,37773	-1,08578	-2,69014
P-value	#DIV/0!	0,62028	0,35122	0,48352	0,20479	0,68939	0,17950	0,36915	0,80175	0,42836
Std.av Grade 1	5,001	0,904	0,560	0,370	0,391	0,468	3,942	0,599	0,728	3,284
Std.av Grade 2	0,000	1,758	0,482	1,279	1,711	0,860	5,981	1,215	0,566	1,808
Max Grade 1	12,643	2,685	6,738	6,818	7,096	7,480	10,648	6,809	5,436	11,263
Min Grade 1	11,008	0,732	5,411	5,821	5,913	6,029	7,488	5,677	3,253	3,816
Max Grade 2	0,000	4,260	7,189	7,249	9,289	7,706	10,598	8,188	4,914	6,222
Min Grade 2	0,000	0,856	6,266	4,707	6,067	5,989	10,102	5,972	3,821	2,858

Table S5a. RT-qPCR re-evaluation of 6 mRNA differential expression (Tumors versus control) with three reference genes. The ΔΔCq method was used to calculate all the fold changes and the Student's t-test (two tailed) was used to calculate all the p-values.

Patient, grade	p63	E-cadherin	PTEN	RICTOR	p53	RUNX1T1
2, II	6,603	3,047	4,454	6,569	5,613	12,186
1, I	4,279	1,274	3,053	4,183	2,309	10,034
3, II	5,028	1,250	2,074	4,271	3,838	10,997
4, I	3,996	2,458	3,979	5,154	2,977	8,930
5, I	11,047	1,399	2,850	4,347	2,752	8,277
6, II	2,662	-1,698	1,861	6,068	3,528	12,226
7, I	6,859	0,387	2,593	5,367	3,848	12,490
8, I	10,271	2,697	3,623	3,696	1,849	10,370
9, I	8,742	1,698	3,678	6,072	3,428	10,024
10, I	4,160	2,214	3,813	5,801	4,023	10,927
11, I	5,606	3,273	4,938	6,774	5,041	9,996
12, I	5,556	1,470	3,423	5,696	3,728	11,401
13, I	3,335	1,909	3,991	6,140	4,369	9,347
15, I	5,271	0,564	2,983	5,211	3,349	9,093
10, N	7,640	5,839	7,310	7,945	5,460	12,132
13, N	8,481	3,870	4,293	4,476	3,150	7,630
15, N	8,877	3,978	5,451	6,392	4,180	7,502
16, NN	11,185	7,723	6,630	5,986	4,421	6,651

Average T	5,96	1,57	3,38	5,38	3,62	10,45
Average N	8,46	5,31	5,87	5,93	4,21	8,58
Fold Change	**5,67074**	**13,42920**	**5,63336**	1,45946	1,50606	**-3,65870**
P-value	**0,04448**	**0,00007**	**0,00004**	0,29833	0,21273	**0,02125**
STD T	2,523	1,271	0,870	0,953	0,999	1,313
STD N	1,938	1,971	1,136	1,249	0,754	1,958
Max T	11,047	3,273	4,938	6,774	5,613	12,490
Min T	2,662	-1,698	1,861	3,696	1,849	8,277
Max N	11,185	7,723	7,310	7,945	5,460	12,132
Min N	7,640	3,870	4,293	4,476	3,150	6,651

Table S5b. Relative expression of mRNA of grade I versus grade II for the mRNAs presented in Table 4 for the differential expression between tumors versus controls. Fold change between grades I and II and the p-values are shown. The ΔΔCq method was used to calculate all the fold changes and the Student's t-test (two tailed) was used to calculate all the p-values.

	**p63**	**E-cadherin**	**PTEN**	**RICTOR**	**p53**	**RUNX1T1**
Average Grade I	6,28	1,76	3,54	5,31	3,42	10,08
Average Grade II	4,76	0,87	2,80	5,64	4,33	11,80
Fold Change, I vs. II	-2,87	-1,86	-1,67	1,25	1,87	3,30
*P-value	0,376	0,299	0,201	0,622	0,175	0,038

* No significance between grade I and II in mRNA expression reported in Table 4 with the exception of RUNX1T1.

Table S6. RNA integrity number (RIN) for used meningioma and Dora (N, NN) samples.

**Patient no.**	**Sample Type**	**RIN Vales**
1	T	9,1
2	T	8.4
3	T	9.1
4	T	7.5
5	T	8.6
6	T	7.7
7	T	7.7
8	T	8.7
9	T	8.8
10	T	9.1
11	T	8.3
12	T	7.4
13	T	8.0
14	T	8.7
15	T	7.8
9	N	6.8
10	N	6.7
13	N	6.6
15	N	6.9
16	NN	7.1

Table S7. RT-qPCR validation-primers for miRNA expression (Exiqon A/S, 2950 Vedbaek, Denmark).

miRNA	Target sequence	Catalog number
hsa-miR-122	UGGAGUGUGACAAUGGUGUUUG	204090
hsa-miR-574-3p	CACGCUCAUGCACACACCCACA	204365
hsa-let-7g	UGAGGUAGUAGUUUGUACAGUU	204565
hsa-miR-17	CAAAGUGCUUACAGUGCAGGUAG	204771
hsa-miR-26b	UUCAAGUAAUUCAGGAUAGGU	204172
hsa-miR-218	UUGUGCUUGAUCUAACCAUGU	204484
hsa-miR-376c	AACAUAGAGGAAAUUCCACGU	204442
hsa-miR-34a	UGGCAGUGUCUUAGCUGGUUGU	204486
hsa-miR-342-3p	UCUCACACAGAAAUCGCACCCGU	204511
hsa-miR-424	CAGCAGCAAUUCAUGUUUUGAA	204736
hsa-miR-143	UGAGAUGAAGCACUGUAGCUC	204190
hsa-miR-130a	CAGUGCAAUGUUAAAAGGGCAU	204658
hsa-miR-152	UCAGUGCAUGACAGAACUUGG	204294
hsa-miR-199a-5p	CCCAGUGUUCAGACUACCUGUUC	204494
hsa-miR-99a	AACCCGUAGAUCCGAUCUUGUG	204521
hsa-miR-148b	UCAGUGCAUCACAGAACUUUGU	204047
hsa-miR-193b	AACUGGCCCUCAAAGUCCCGCU	204226
hsa-miR-451	AAACCGUUACCAUUACUGAGUU	204734
hsa-miR-21	UAGCUUAUCAGACUGAUGUUGA	204230
hsa-miR-200a	UAACACUGUCUGGUAACGAUGU	204707
hsa-miR-191	CAACGGAAUCCCAAAAGCAGCUG	204306
hsa-miR-16	UAGCAGCACGUAAAUAUUGGCG	204409
hsa-let-7a	UGAGGUAGUAGGUUGUAUAGUU	204775

Table S8. Assay ID for mRNA targets (Life Technologies Corporation, Carlsbad CA, USA).

**Target mRNA**	**Catalog number**	**Assay ID**	**Gene group**
Cyclin G1	4351372	Hs01063261_g1	Cyclin-dependent protein kinases (CDKs)
p53	4331182	Hs01034249_m1	Tumor suppressor gene
p63	4331182	Hs00978343_m1	Tumor suppressor gene, epithelial cell cells biomarker.
RUNX1T1	4331182	Hs00231702_m1	Runt-related transcription factor 1; translocated to, 1 (cyclin D-related)
E2F5	4331182	Hs00231092_m1	E2F family of transcription factors
PTEN	4331182	Hs02621230_s1	Tumor suppressor gene; Phosphatase and tensin homolog
Rictor	4331182	Hs00380903_m1	Cell growth
ALK7 (*ACVR1C*)	4331182	Hs00899854_m1	TGFB receptor; activin A receptor
SIRT1	4331182	Hs01009005_m1	Protein Deacteylation. Nicotinamide adenosine dinucleotide (NAD)-dependent deacetylase
mTOR	4331182	Hs00234508_m1	Conserved serine/threonine kinase (signal Transduction)
E-Cadherin (*CDH1*)	4331182	Hs01023894_m1	Epithelial tissue biomarker
GAPDH	4331182	Hs03929097_g1	**Reference gene**
Beta-Actin- (*ACTB*)	4331182	Hs01060665_g1	**Reference gene**
HPRT1- (hypoxanthine phosphoribosyltransferase 1)	4331182	Hs02800695_m1	**Reference gene**

Figure S1. miRNA expression in meningioma relative to normal control. Expression values are given as ΔCq values; hence, lower ΔCq values are equal to higher expression. Standard deviation is shown as vertical lines.

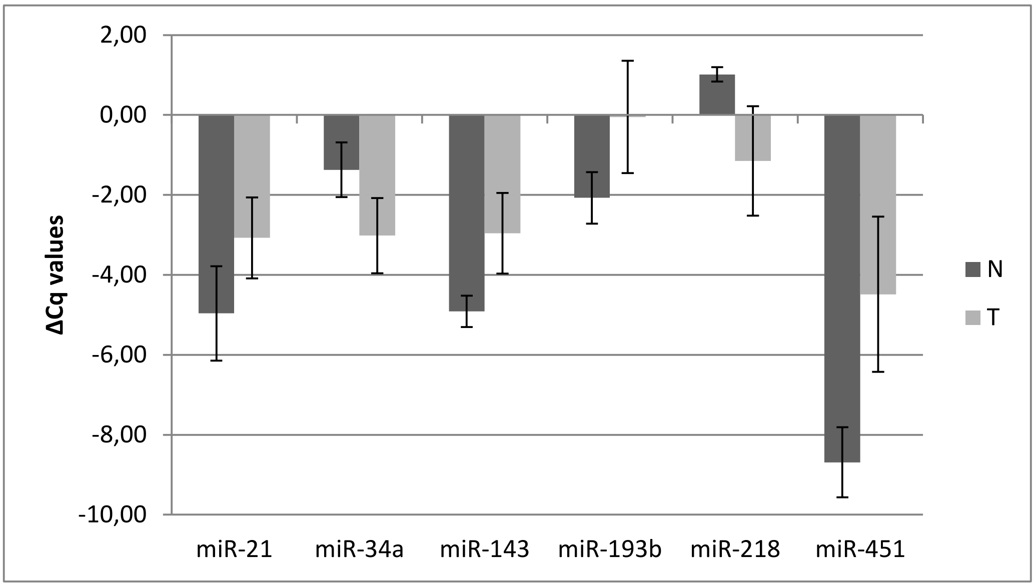

Figure S2. mRNA expression in meningioma relative to normal control. Expression values are given as ΔCq values; hence, lower ΔCq values are equal to higher expression. Standard deviation is shown as vertical lines.

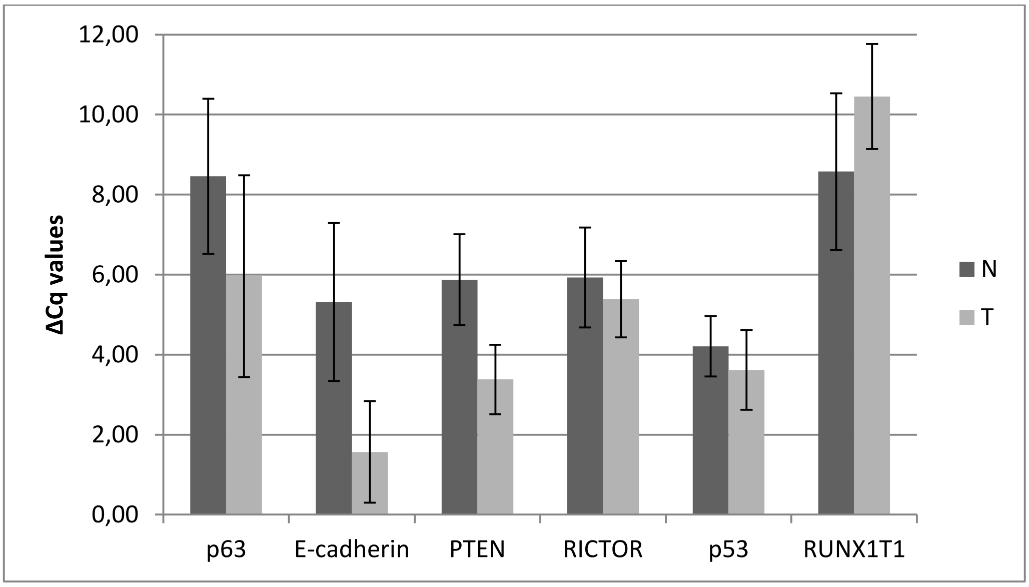

Supplement: Supplementary file 1 [file cancers-08-00031-s001.pdf]
